# *Prevotella* are major contributors of sialidases in the human vaginal microbiome

**DOI:** 10.1073/pnas.2400341121

**Published:** 2024-08-26

**Authors:** Paula Pelayo, Fatima A. Hussain, Caroline A. Werlang, Chloe M. Wu, Benjamin M. Woolston, Claire M. Xiang, Lindsay Rutt, Michael T. France, Jacques Ravel, Katharina Ribbeck, Douglas S. Kwon, Emily P. Balskus

**Affiliations:** ^a^Department of Chemistry and Chemical Biology, Harvard University, Cambridge, MA 02138; ^b^Ragon Institute of Massachusetts General Hospital, Massachusetts Institute of Technology and Harvard, Massachusetts General Hospital, Cambridge, MA 02139; ^c^Department of Biological Engineering, Massachusetts Institute of Technology, Cambridge, MA 02139; ^d^Department of Chemical Engineering, Northeastern University, Boston, MA 02115; ^e^Institute for Genome Sciences, University of Maryland School of Medicine, Baltimore, MD 21201; ^f^HHMI, Harvard University, Cambridge, MA 02138

**Keywords:** sialidase, mucin, bacterial vaginosis, vaginal microbiome, *Prevotella*

## Abstract

Elevated bacterial sialidase activity in the female genital tract is strongly associated with poor health outcomes including preterm birth and bacterial vaginosis (BV). These negative effects may arise from sialidase-mediated degradation of the protective mucus layer in the cervicovaginal environment. Prior biochemical studies of vaginal bacterial sialidases have focused solely on the BV-associated organism *Gardnerella vaginalis*. Despite their implications for sexual and reproductive health, sialidases from other vaginal bacteria have not been characterized. Here, we show that vaginal *Prevotella* species produce sialidases that possess variable activity toward mucin substrates. The sequences of sialidase genes and their presence are largely conserved across clades of *Prevotella* from different geographies, hinting at their importance globally. Finally, we find that *Prevotella* sialidase genes and transcripts, including those encoding mucin-degrading sialidases from *Prevotella timonensis*, are highly prevalent and abundant in human vaginal genomes and transcriptomes. Together, our results identify *Prevotella* as a critical source of sialidases in the vaginal microbiome, improving our understanding of this detrimental bacterial activity.

## Significance

Sialidase activity in the vaginal microbiome is increased in bacterial vaginosis and strongly associated with other adverse health outcomes. Sialidase enzymes release sialic acid from host-derived glycans in the vaginal environment, altering their structures and functions. However, biochemical studies of vaginal bacterial sialidases have been limited to one genus, *Gardnerella*. In this work, we identify and characterize multiple sialidase enzymes in vaginal bacteria of the genus *Prevotella*, including an enzyme active toward human mucin. We find that genes and transcripts encoding *Prevotella* sialidases are more prevalent and abundant in vaginal microbial communities than those from *Gardnerella*. Our work highlights *Prevotella* bacteria as an underappreciated source of sialidase transcripts in metatranscriptomes with important implications for our understanding of sialidase producers in the vaginal ecosystem.

The microbial community that inhabits the human vagina (the vaginal microbiome) is important for sexual and reproductive health. The composition of the vaginal microbiome can differ substantially between individuals (1). While beneficial health outcomes have been associated with *Lactobacillus*-dominated vaginal microbiomes, more diverse communities containing anaerobic bacteria have been associated with increased risk for preterm birth, bacterial vaginosis (BV) (2), and HIV acquisition (3). Despite these strong connections to health, the specific mechanisms by which vaginal bacteria influence the host are poorly understood. An understanding of the vaginal bacterial functions most strongly linked to adverse health outcomes is needed to enhance our understanding of this microbial community and guide the design of vaginal microbiome-targeted therapeutics.

**Fig. 1.**
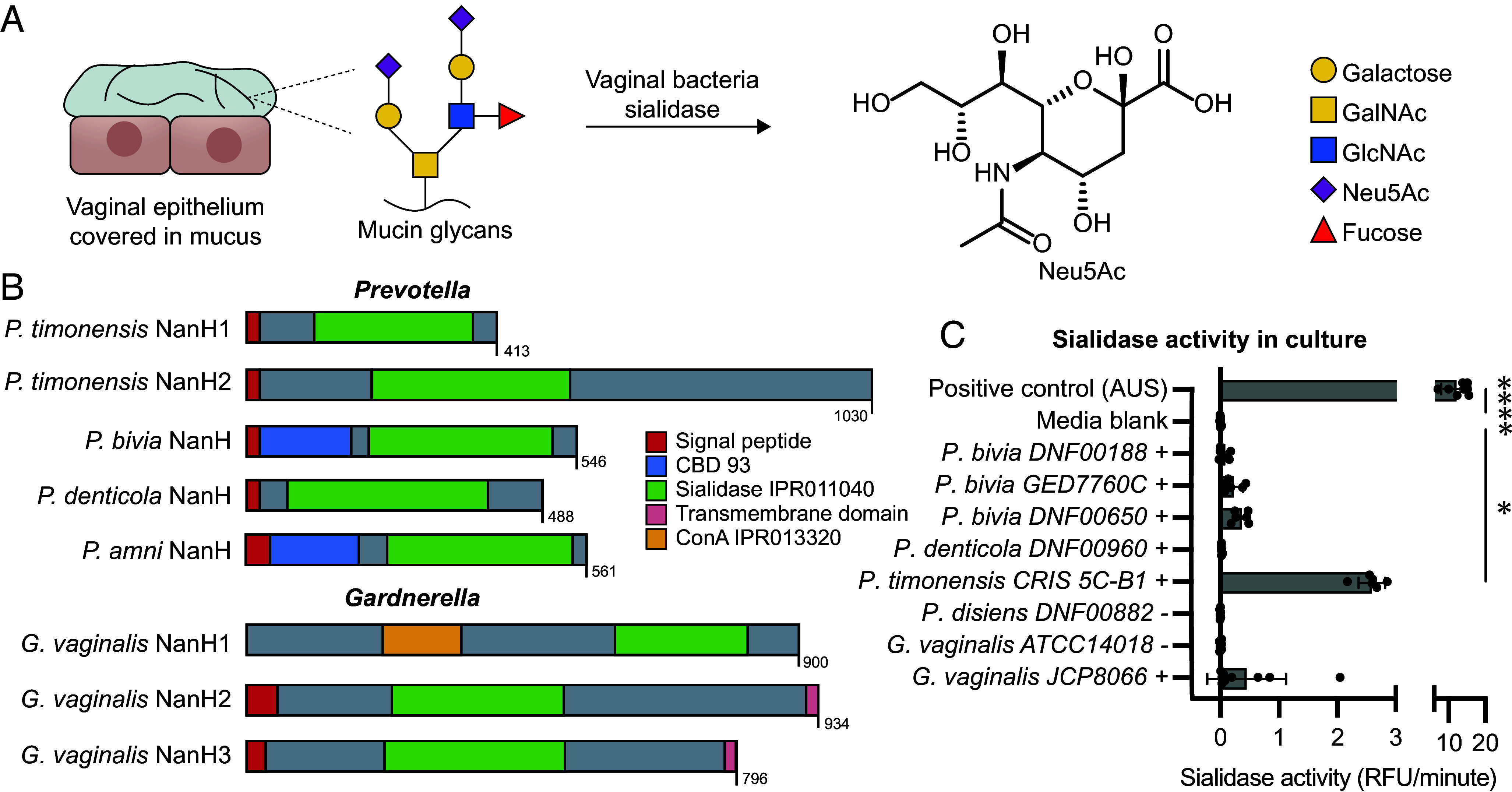
Vaginal *Prevotella* species encode diverse sialidases. (*A*) Vaginal bacteria sialidase enzymes are hypothesized to remove sialic acids (such as Neu5Ac) from the mucin glycans that comprise the protective mucus layer covering vaginal epithelial cells. (*B*) *Prevotella timonensis* CRIS 5C-B1*, Prevotella bivia* DNF00188, *Prevotella denticola* DNF00960, and *Prevotella amnii* CRIS21A-A encode proteins with predicted sialidase domains. Previously characterized *Gardnerella* sialidases’ protein domains are also displayed for comparison. Individual colors represent different domains and features: signal peptide (red), sialidase domain IPR011040 (green), and carbohydrate binding domain 93 (blue). The amino acid length is displayed for each bar. (C) *Prevotella* isolates display sialidase activity that correlates with the presence of candidate sialidase genes, indicated by +/–. Bacterial isolates were cultured in PYGT media containing 1 % glucose and 10 % horse serum. Sialidase activity was measured in whole culture samples. Positive control represents the full hydrolysis of 4-methylumbelliferyl N-acetyl-α-D-neuraminic acid (4-MU-Neu5Ac) by *Arthrobacter ureafaciens* sialidase (AUS). *Gardnerella vaginalis ATCC14018* is indicated as a (–) because it encodes NanH1 but not NanH2 or NanH3. *G. vaginalis JCP8066* encodes NanH1 and NanH3. Data represent the average ± SD of >5 biological replicates. Significance was assessed using one-way ANOVA followed by the multiple comparisons test, ****P < 0.0001, *P < 0.05. Significance values represent comparison to the media blank.

Elevated sialidase activity in vaginal fluid is associated with increased risk of preterm birth (4, 5) and is a common feature of BV (6–8). Sialidases are glycoside hydrolase (GH) enzymes that hydrolyze terminal sialic acids (such as *N*-acetyl-neuraminic acid, Neu5Ac) from glycans present on proteins and lipids (Fig. 1*A*). Multiple, varied sources of sialic acid are found in the female genital tract. Sialic acids are incorporated into the terminal end of mucin glycans, which are prominent components of the mucus layer that covers cervical and vaginal epithelial cells. Cervical mucus secretions contain gel-forming mucins (MUC5B, MUC5AC, and MUC6) as well as transmembrane mucins (MUC16 and MUC1), with MUC5B being the most abundant (9). Immunoglobulins in the cervical mucus (e.g., IgG) are also sialylated, and sialic acids are important for antibody regulation and function (10, 11). Changes in mucus properties are linked to preterm birth risk (12), suggesting that the activity of sialidases on mucins could have implications for mucus function and host health.

Sialidase activity in the female genital tract has long been attributed to the anaerobic vaginal bacterium *G. vaginalis*, which can encode up to three different sialidases (NanH1, NanH2, and NanH3) (13). The *nanH1* gene (previously sialidase A), which is highly prevalent in Gardnerella-positive vaginal samples (13–15), was the first sialidase gene identified (16) in this organism and was assumed to be responsible for sialidase activity without biochemical characterization of the corresponding protein. However, recent biochemical characterization of this enzyme showed it has little activity toward the sialidase substrate 4-methylumbelliferyl *N*-acetyl-α-D-neuraminic acid (4-MU-Neu5Ac) (13). Lacking a signal peptide, NanH1 is also predicted to be intracellular, making it unlikely to interact with mucin. In contrast, the extracellular enzymes NanH2 and NanH3 display greatly increased activity toward 4-MU-Neu5Ac and can efficiently remove sialic acids from bovine submaxillary mucin (BSM) (13, 17, 18).

Accumulating evidence suggests sialidase activity may be more widespread among vaginal bacteria. For example, several studies have found that *Prevotella bivia* (7)*, P. timonensis* (also called *Hoylesella timonensis*), and *Bacteroides fragilis* strains isolated from vaginal samples possess sialidase activity (6, 19, 20). A recent analysis of transcriptomes found that *Prevotella* species express a large fraction of predicted sialidase genes (annotated by the Carbohydrate Active enZymes database, CAZy) in diverse vaginal samples (21). *Prevotella* strains also have been isolated from the upper reproductive tract (22) and their abundance in the vagina is correlated with preterm birth (23, 24). Persistent sialidase activity in women with recurrent BV has also previously been associated with *P. bivia* (6). *P. timonensis* isolates can alter the endometrial epithelial barrier and induce mucin expression (MUC3 and MUC4) in a 3D epithelial cell model (20). *P. timonensis* presence in early pregnancy is also a predictor for higher risk of preterm birth (2). Despite the intriguing links between sialidase activity, preterm birth, and *Prevotella*, putative sialidases from these bacteria have not been biochemically characterized and their activities remain poorly understood.

Here, we investigate sialidases from vaginal *Prevotella* species as a first step toward understanding their contributions to sialidase activity in the female genital tract. We initially biochemically characterize sialidases from three *Prevotella* species in vitro, observing unexpected differences in activity toward mucin substrates, including the human mucin MUC5B. We use comparative genomics to demonstrate that genes encoding sialidases are widely distributed across vaginal *Prevotella* isolates from the United States and South Africa and their sequences and presence are largely conserved within phylogenetic groups. Finally, through analysis of human vaginal metagenomes (MG) and metatranscriptomes (MT), we find that *P. timonensis* sialidase-encoding genes and transcripts are more prevalent than sialidase-encoding genes from other vaginal bacteria, including *G. vaginalis*. These findings reveal *Prevotella* bacteria as important, underappreciated contributors to sialidase activity in the human vaginal microbiome and highlight a need to understand the biological roles of these enzymes in the vaginal environment.

## Results

### Vaginal *Prevotella* Species Encode Diverse Sialidases.

To identify candidate sialidase genes in vaginal *Prevotella*, we initially analyzed seven publicly available genomes of from vaginal isolates of *P. bivia, P. amnii, P. denticola, P. disiens*, and *P. timonensis* from BEI Resources (Biodefense and Emerging Infections). Sequences of biochemically characterized sialidases from *Gardnerella* (NanH2 and NanH3) were used as query. We found five genes encoding candidate sialidases in four *Prevotella* species, *P. bivia* DNF00188 (*PbnanH*), *P. amnii* CRIS21A-A (*PananH*), *P. denticola* DNF00960 (*PdnanH*), and *P. timonensis* CRIS 5C-B1 (*PtnanH1* and *PtnanH2*) (*SI Appendix*, Fig. S1). All five proteins contain the catalytic active site residues characteristic of the GH33 family of sialidases (*SI Appendix*, Fig. S2 and Table S5). All sequences also contain a predicted signal peptide, indicating they are likely extracellular, and a sialidase catalytic domain (IPR011040) (Fig. 1*B*) with the predicted β-propeller fold characteristic of these enzymes (*SI Appendix*, Figs. S3–S5). *Pb*NanH and *Pa*NanH also contain a predicted carbohydrate-binding domain 93, which is typically found in sialidases from commensal gut *Bacteroides* (25). We found that *P. timonensis* encodes two sialidases of different lengths (*Pt*NanH1 413 amino acids; *Pt*NanH2 1,030 amino acids) and AlphaFold (26) structure prediction indicates *Pt*NanH2 may contain four additional domains of unknown function which are not homologous to structurally characterized protein domains (*SI Appendix*, Fig. S5*B*).

We next cultured available vaginal *Prevotella* isolates in PYGT medium with 1 % glucose and 10 % horse serum and tested for sialidase activity in culture using the fluorescent substrate 4-MU-Neu5Ac. Notably, of all strains tested, *P. timonensis* CRIS 5C-B1 consistently had the highest sialidase activity (Fig. 1*C*). Sialidase activity in *G. vaginalis* JCP8066, which encodes NanH3, was detectable but variable across different experiments. Other *Prevotella* strains, such as *P. bivia* (24), encoding sialidases had low but detectable activity. No activity was observed for a *P. disiens* DNF00882 isolate lacking a sialidase homolog. Together, this work further confirmed the presence of sialidase activity in vaginal *Prevotella* strains and identified candidate sialidase enzymes for biochemical studies.

### *Prevotella* Sialidases Are Active and Susceptible to Inhibition.

**Fig. 2.**
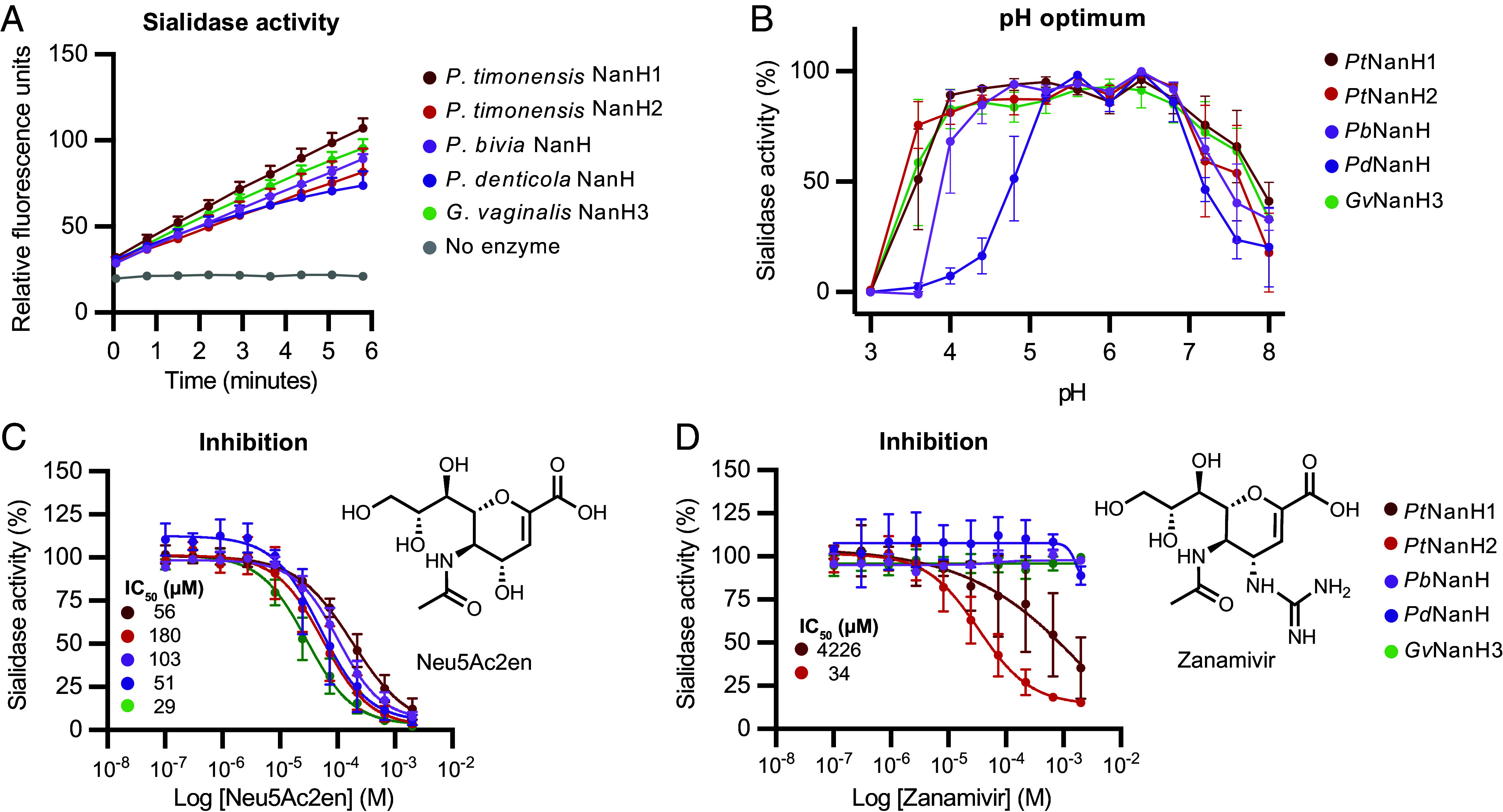
*Prevotella* sialidases are active at varying pH levels and are inhibited by small molecules. (*A*) Purified sialidase enzymes hydrolyze 4-MU-Neu5Ac, indicating they possess sialidase activity. For each time point, 2.5 nM enzyme was incubated with 200 µM 4-MU-Neu5Ac in sodium acetate buffer, pH 5.5 at 37 °C. Data represent the average + SEM of three independent experiments. (*B*) Sialidase activity at varying pH. 200 µM 4-MU-Neu5Ac was prepared in 0.1 M citric acid/0.2 M phosphate buffer at pH values ranging from 3 to 8. Data represent the average ± SEM of three independent experiments. Inhibitory activity of (*C*) Neu5Ac2en and (*D*) Zanamivir toward *Prevotella* and *Gardnerella* sialidases (IC_50_ values can be found in *SI Appendix*, Table S8). Purified sialidases were preincubated with inhibitor for 15 min before adding 4-MU-Neu5Ac to determine activity. Data represent the average ± SEM of three independent experiments.

To determine whether the predicted *Prevotella* sialidase genes encoded active sialidase enzymes, we expressed the putative sialidases from *P. timonensis* CRIS 5C-B1*, P. bivia* DNF00188, and *P. denticola* DNF00960 in *Escherichia coli* and purified them for in vitro biochemical characterization (*SI Appendix*, Fig. S6). We found all purified *Prevotella* sialidases were active toward 4-MU-Neu5Ac (Fig. 2*A*). The kinetics of vaginal bacterial sialidases have not yet been examined; we therefore determined the Michaelis–Menten kinetic parameters of the *Prevotella* sialidases and *Gv*NanH3. We found their turnover rates *k*_cat_ (110.46 to 149.33 s^−1^) and catalytic efficiencies k_cat_/K_m_ (0.51 to 1.83 × 10^6^ s^−1^ M^−1^) (*SI Appendix*, Table S6) comparable to those of other previously characterized bacterial sialidases (*SI Appendix*, Table S7).

Health-associated *Lactobacillus*-dominated vaginal communities have a pH < 4.5^1^, while *Prevotella* species are typically found in more diverse vaginal communities which have a pH > 4.5. We therefore examined the pH dependence of the *Prevotella* sialidases and *Gv*NanH3. Measuring the activity of the *Prevotella* sialidases toward 4-MU-Neu5Ac over a pH range of 3.0 to 8.0 revealed that all sialidases had maximal activity above pH 4.5 (Fig. 2*B*). Interestingly, among the tested enzymes *Pt*NanH1, *Pt*NanH2, and *Gv*NanH3 displayed activity below pH 4 while *Pd*NanH was only active above pH 4.8.

To further confirm the functional assignment of these enzymes, we examined their susceptibility to various commercially available sialidase inhibitors. All enzymes were inhibited by the well-characterized, broad-spectrum sialidase inhibitor, Neu5ac2en (Fig. 2*C* and *SI Appendix*, Table S8) (27). Unexpectedly, the viral sialidase inhibitor Zanamivir (Relenza) was effective toward *P. timonensis* CRIS 5C-B1 sialidases *Pt*NanH1 and *Pt*NanH2 but no other enzymes tested (Fig. 2*D*). Inhibition of sialidase activity by Neu5Ac2en and Zanamivir was also observed in *P. timonensis* cultures (*SI Appendix*, Fig. S8). Overall, these results further support the characterization of these enzymes as sialidases, with the variable activity of Zanamivir suggesting potential differences in structure and function between the *P. timonensis* sialidases and those from other *Prevotella* species.

### *Prevotella* Sialidases Have Variable Activity Toward Mucin Glycoproteins.

**Fig. 3.**
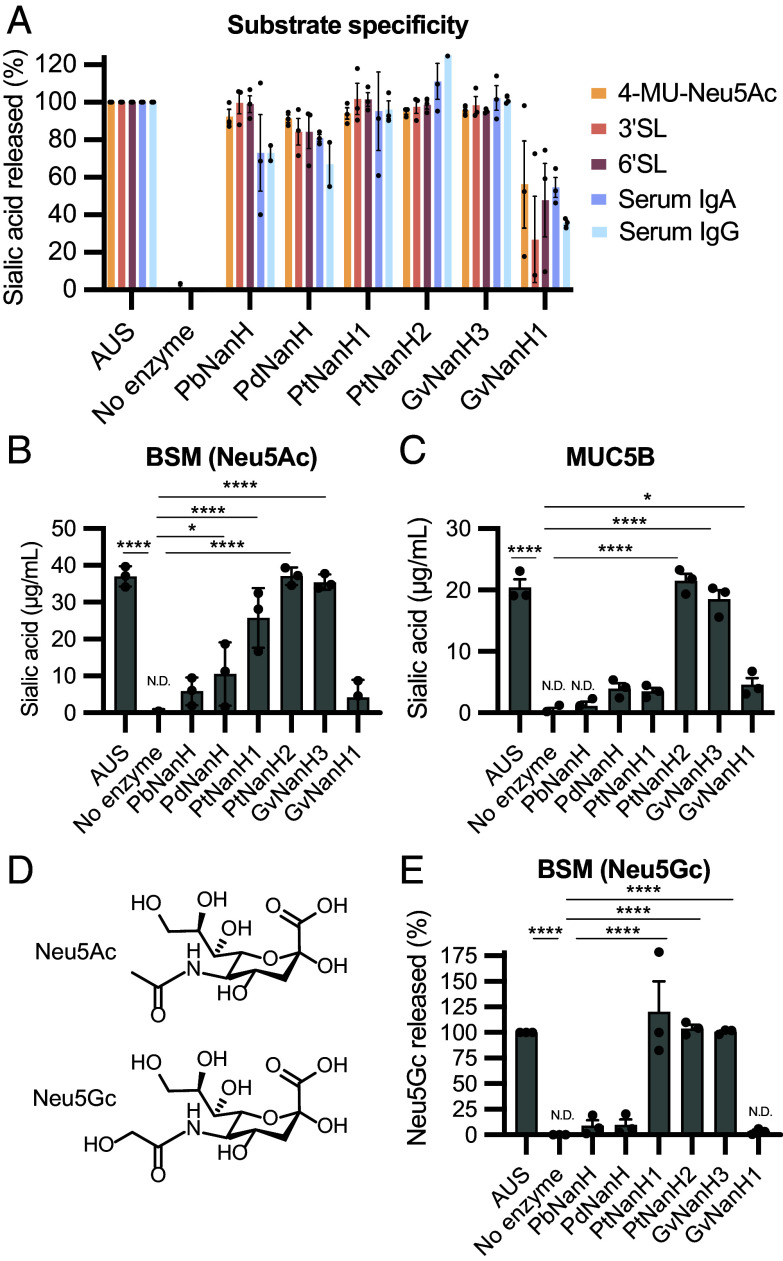
*Prevotella* sialidases have varied substrate specificities. (*A*) Quantification of sialidase activity toward different substrates: 4-MU-Neu5Ac, 3′SL and 6′SL, human serum IgG, and human serum IgA. Purified sialidase enzyme (20 to 100 nM) was incubated with each substrate in 20 mM sodium acetate buffer pH 5.5 for 2 h at 37 °C. AUS was used a positive control to release the total sialic acid from each substrate (31). Sialidase activity toward (*B*) BSM and (*C*) purified human salivary MUC5B. (*D*) Structures of Neu5Ac and *N*-glycolylneuraminic acid (Neu5Gc). Data represent the average ± SEM of three independent experiments. N.D. represents values were not detected below the limit of detection. (*E*) Sialidase activity toward Neu5Gc from BSM. Data represent the average ± SEM of three independent experiments. Significance was assessed using one-way ANOVA followed by the multiple comparisons test, ****P < 0.0001, *P < 0.05. Significance values represent comparison to the no enzyme control.

Sialic acids are incorporated into a variety of potential substrates, including mucin glycans, via α2-3′ and α2-6′ linkages. We sought to determine whether *Prevotella* sialidases had differences in linkage and substrate preferences by testing their activity toward a panel of six Neu5Ac-containing substrates (Fig. 3*A*). We incubated purified sialidases with α2-3′-sialyllactose (3′SL), α2-6′-sialyllactose (6′SL), BSM, human salivary MUC5B, immunoglobulins A (IgA), and immunoglobulins G (IgG), and quantified Neu5Ac released by each enzyme in an endpoint liquid chromatography–mass spectrometry (LC–MS) assay. Salivary MUC5B and BSM were used because they share certain sialylation patterns and linkages [α(2,3) Neu5Ac bound to galactose and α(2, 6) Neu5Ac bound to GalNAc] with cervical MUC5B (*SI Appendix*, Fig. S9). Although the precise sialylation patterns of cervical immunoglobulins are unknown, we used commercially available IgG and IgA, which have been previously used as substrates for vaginal bacterial sialidase activity (17, 28). We found that all sialidases released sialic acid from 3′SL and 6′SL substrates, indicating these sialidases do not have a strong preference for linkage type (Fig. 3*A*). All sialidases tested released sialic acid from IgA and IgG, the predominant immunoglobulins in cervical mucus (29, 30) (Fig. 3*A*). Unexpectedly, the *Prevotella* sialidases differed in their activity toward mucin substrates. While *Pt*NanH2 and *Gv*NanH3 efficiently removed sialic acid from both BSM and human MUC5B, *Pt*NanH1, *Pb*NanH, and *Pd*NanH had reduced activity (30 to 40%) toward BSM and little to no activity toward MUC5B (Fig. 3 *B* and *C*). The variable activity of vaginal bacterial sialidases toward the human mucin MUC5B is unexpected given the potential links between mucin degradation and negative health outcomes.

We next sought to explore whether *Prevotella* sialidases could release other forms of sialic acid from BSM, which contains diverse sialic acid structures including Neu5Ac and Neu5Gc, Neu5,7Ac_2_, and Neu5,9Ac_2_. *Pb*NanH resembles Sialidase26 (66% amino acid ID), recently characterized from the human gut MG (32), which has preferential activity toward Neu5Gc (Fig. 3*D*). While the presence of Neu5Gc in cervicovaginal mucins is unclear, this finding prompted us to assess whether vaginal bacterial sialidases could release Neu5Gc from BSM. Consistent with our previous assay results, *Pb*NanH and *Pd*NanH did not release Neu5Gc from BSM (Fig. 3*E*). Among the sialidases observed previously to release Neu5Ac from BSM, only *Pt*NanH1, *Pt*NanH2, and *Gv*NanH3 also released Neu5Gc from this substrate, further indicating these enzymes are more promiscuous than the other sialidases (Fig. 3*E*). Together, the results of this substrate survey demonstrate *Prevotella* sialidases can release sialic acid from substrates resembling those found in the cervicovaginal environment. Notably, the *P. timonensis* CRIS 5C-B1 *Pt*NanH2 possess similar reactivity to the previously reported active *Gardnerella* sialidase *Gv*NanH3^13^.

### Sialidases Are Conserved in *Prevotella* Isolates From Different Geographies.

**Fig. 4.**
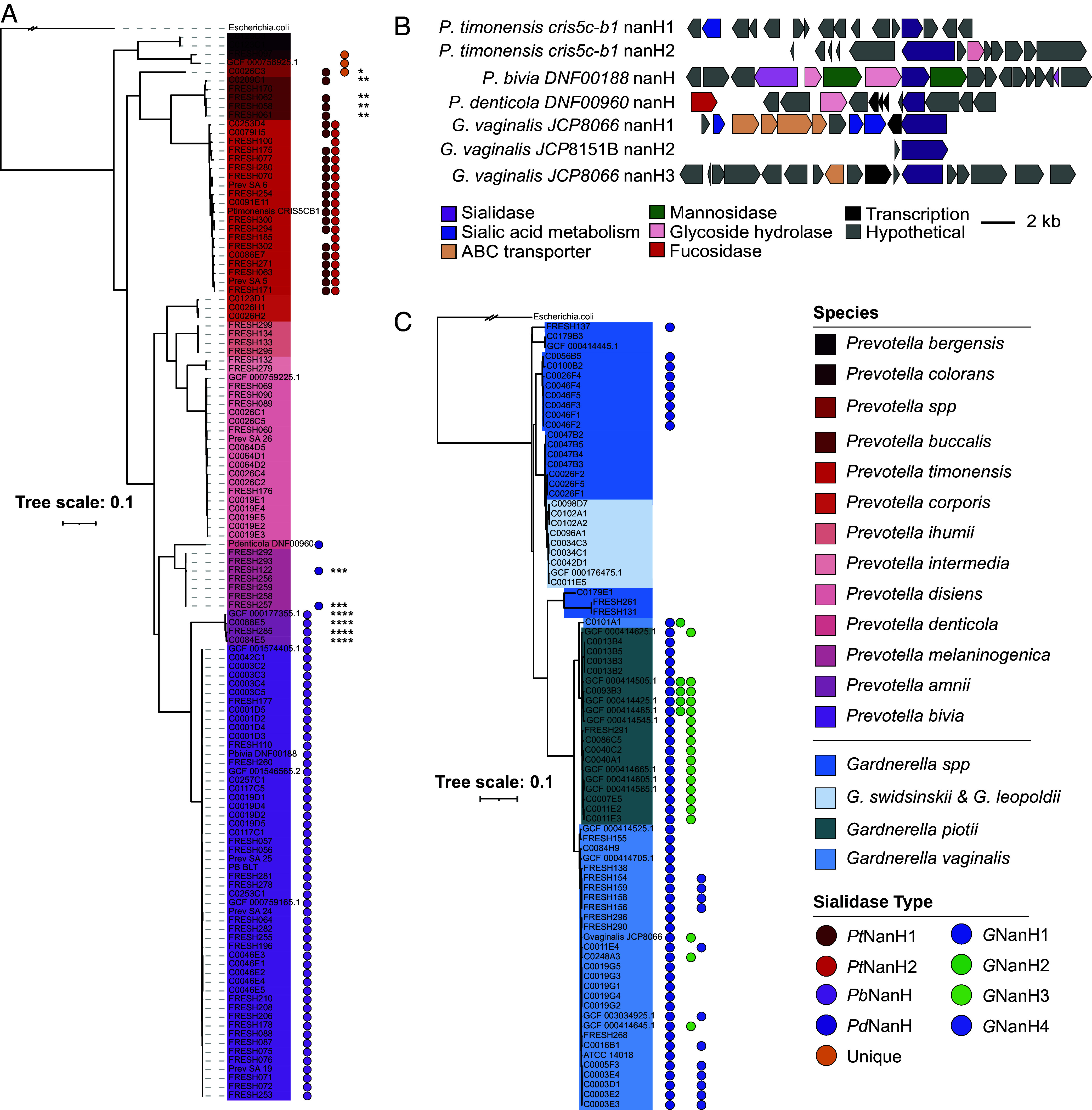
Sialidases are widely distributed across *Prevotella* and *Gardnerella* isolates obtained from the United States and South African vaginal samples. Phylogenetic trees of vaginal isolates from the Vaginal Microbiome Research Consortium (N. American) and FRESH (S. African) studies. (*A*) Sialidase genes present in individual *Prevotella* isolate genomes (gene presence is shown as a filled circle). (*B*) Genome neighborhoods of *Prevotella* and *Gardnerella* sialidases. (*C*) Sialidase genes present in individual *Gardnerella* isolate genomes. The phylogeny is based on 49 concatenated ribosomal proteins and serves as a proxy for the core genome. The scale bar indicates nucleotides substitutions per site. Each genome was searched using the sialidase protein alignment with HMMER (version 3.1b2). See *SI Appendix*, Figs. S13 and S15 for the unrooted *Prevotella* and *Gardnerella* phylogenetic trees. * = 56% amino acid ID to *Pt*NanH1, ** = 61% AA ID to *Pt*NanH1, *** = 78% amino acid ID to *Pd*NanH, **** = 70% amino acid ID to *Pb*NanH. The orange filled circle indicates a sialidase that is unique from the *Prevotella* sialidases characterized here.

Having characterized the activities of the *Prevotella* sialidases, we next sought to determine the prevalence of sialidase genes across diverse strains of *Prevotella* and compare their distribution to that of other characterized and predicted vaginal bacterial sialidase genes. Employing an HMM-based approach using the sialidases characterized in this work and other GH33 sialidases from the CAZy database, we searched whole genomes from over 1,000 bacterial strains isolated from vaginal samples from cohorts based in the United States and South Africa (*SI Appendix*, Figs. S10 and S11). We observed that sialidase genes in *P. timonensis* and *P. bivia* are highly conserved within isolates of these species and that the encoded proteins share high amino acid ID. All 53 *P. bivia* isolates encode a highly similar *Pb*NanH sialidase (>98% amino acid ID) regardless of the geography of origin. Similarly, all 21 *P. timonensis* isolates encode *Pt*NanH2 (>93% amino acid ID) and 19/21 encode *Pt*NanH1 (>94% amino acid ID) (Fig. 4*A*). Additionally, close relatives of *P. timonensis* also encode distantly related sialidase genes. A vaginal *P. buccalis* isolate encodes a sialidase with 61% amino acid ID to *Pt*NanH1 but lacking a signal peptide. *Prevotella colorans *FRESH097** and *P. sp16 *C0026C3** encode a putative sialidase distinct from the ones characterized here with only 9% and 23% amino acid ID to *Pt*NanH1, respectively, and both contain signal peptides (*SI Appendix*, Fig. S12). Interestingly, we did not detect any genes encoding sialidases in clades of *P. disiens*, *Prevotella intermedia, Prevotella ihumii,* or *Prevotella corporis*, and we did not find additional examples of vaginal *P. denticola* in these isolate collections. Inspecting the gene neighborhoods of these *Prevotella* sialidase genes revealed they are usually found near other genes encoding GH enzymes and are not usually colocalized with sialic acid metabolism genes, except for *P. timonensis nanH1* which is near *nanE* (UDP-N-acetylglucosamine 2-epimerase) (Fig. 4*B*). Notably, sialidase activity experiments with additional strains of *P. timonensis, P. bivia, and P. amnii* showed consistent levels of sialidase activity across individual species, suggesting the presence of sialidase genes may be predictive of function (*SI Appendix*, Fig. S14).

*Gardnerella* isolates varied in their sialidase content based on clade, as reported previously (33). *Gardnerella swidsinskii* and *Gardnerella leopoldi* did not encode any sialidases, while the other *Gardnerella* clades encode *GnanH1* (61/73). Notably, the active sialidases *GnanH2* (5/73) and *GnanH3 (*20/73) are much less prevalent than *GnanH1* and are unevenly distributed across *G. vaginalis* and *Gardnerella piotii* strains (Fig. 4C). Interestingly, this analysis revealed variants of *GnanH1* exist among different *Gardnerella* clades. For example, a variant of NanH1 found in *Gardnerella* isolates from the United States shares 82 % amino acid ID with NanH1 found in *Gardnerella* strains from South Africa. The *G. vaginalis *JCP8066** NanH3 enzyme used in this study is homologous to *G. piotii* NanH3 (92% amino acid ID). We also identified an additional candidate sialidase, called NanH4, in 12 *G. vaginalis* isolates; however, NanH4 may be inactive since we verified (via BLAST) it is found in *G. vaginalis *ATCC 49145*,* which does not demonstrate sialidase activity in culture, and is predicted to be intracellular (13).

We also found putative GH33 sialidases encoded in diverse vaginal isolates from the genera *Bacteroidales, Bacteroides, Bifidobacterium, Corynebacterium (Actinomycetia*), and *Streptococcus* (*SI Appendix*, Figs. S10 and S11). *Streptococcus agalactiae* encodes a sialidase that is >98% ID at the amino acid level to Group B *Streptococcus* NonA (34), an inactive homolog of the *Streptococcus pneumoniae* NanA sialidase. There was no sialidase present in its sister clade *Streptococcus anginosus*. Overall, this genome survey increases knowledge of vaginal bacterial sialidases by broadening the search to a wide variety of species and geographies. The substantial variability we observe in sialidase gene presence across certain vaginal bacterial species highlights a need to directly identify these enzymes in vaginal microbiomes rather than infer their presence from phylogenetic information.

### *P. timonensis* Sialidase Genes and Transcripts Are Prominent in Vaginal Microbiomes.

**Fig. 5.**
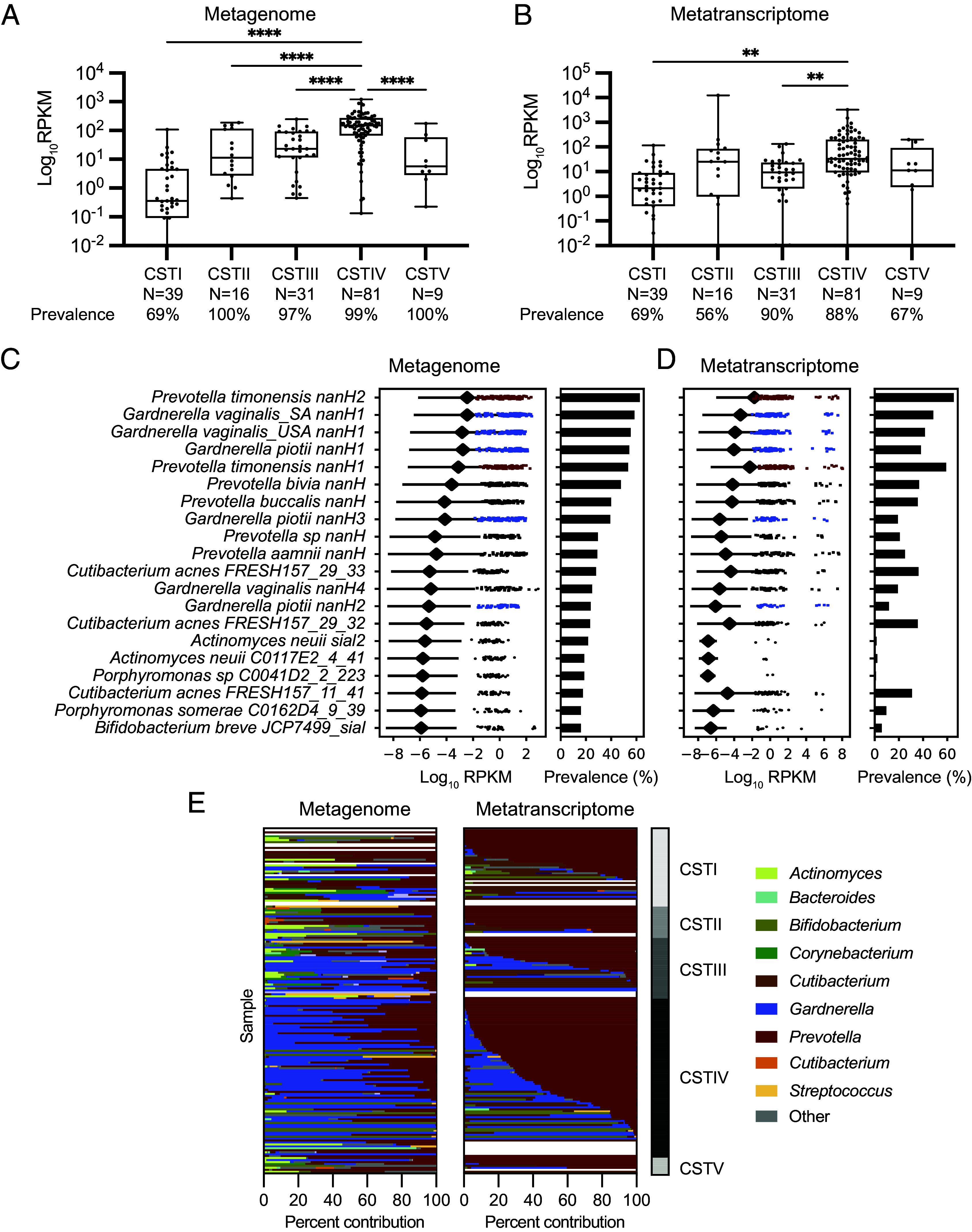
*Prevotella* sialidase genes and transcripts are prevalent across vaginal community state types. (*A*) Total sialidase abundance and prevalence in paired MG and (*B*) MT samples (n = 176). Abundance was determined by Diamond blastX and displayed as reads per kilobase million (RPKM). The cutoff for sialidase prevalence is sialidase reads > 0. Significance was assessed by a one-way ANOVA followed by a Brown–Forsythe and Welch test, **P < 0.0001, **P < 0.01. (*C* and *D*) The abundance and prevalence of specific sialidase genes in all available samples (MG; n = 193, MT; n = 188). The figure displays sialidase genes with >15 % prevalence in MG samples. Abundance values were calculated by adding RPKM to a pseudocount (1E8). The diamond indicates the average sialidase expression across all samples ± SD. See complete list of representative sialidase genes in *SI Appendix*, Fig. S17. (*E*) Contribution of sialidase abundance within individual vaginal samples across MG and MT. Paired MG and MT were used to investigate the relative contribution of several vaginal sialidases. Stacked bar graphs are aligned to pair the corresponding MG and MT sample.

We next identified the genes encoding biochemically characterized *Prevotella* and *Gardnerella* sialidases, as well as the additional putative sialidases from our genome survey, in human vaginal microbiomes. To quantify the differential abundance and prevalence of sialidase-encoding genes and transcripts, we performed translated nucleotide sequence searches in paired MG and MT from vaginal samples sequenced by France et al. (21) (Fig. 5*A*). These samples were collected from 39 reproductive-aged, nonpregnant women at up to 5 timepoints over the span of 10 wk. We analyzed all the samples individually and paired the MG and MT samples (n = 176) with the associated subject metadata to examine association of sialidase gene expression with community state type (CST), including *L. crispatus*–dominated (CSTI), *L. gasseri*–dominated (CSTII), *L. iners*–dominated (CSTIII), diverse anaerobic (CSTIV), and *L. jensenii*–dominated (CSTV) communities. We expected to find higher levels of sialidase gene expression in CSTIV communities compared to other CSTs since sialidase activity is detected in subjects with BV and is typically absent from *Lactobacillus*-dominated samples (6, 35). Unexpectedly, sialidase genes and transcripts were detected in a high percentage of all CSTs (69 to 100% of MGs and 56 to 90% of MTs) with the lowest prevalence found in CSTI samples. However, we found that CSTIV MGs encode a greater abundance of sialidase genes compared to all other CSTs (Fig. 5*A*). We also found that CSTIV MTs have significantly higher sialidase gene expression than MTs from CSTI and CSTIII samples (Fig. 5*B*).

The prevalence of individual sialidase genes varied across all vaginal MGs: *Gardnerella* sialidase genes were detected in 23%–58% of samples, *P. bivia* (*PbnanH* 47% of samples), *P. amnii* (*PananH* 29% of samples), and *P. timonensis* sialidase genes (*PtnanH1* 51% and *PtnanH2* 62% of samples) were identified at similar frequencies, while the sialidase gene from *P. denticola* was not detected (0%), likely because this organism is rarely found in the vaginal microbiome (Fig. 5*C*). In vaginal MTs, *P. timonensis* sialidase transcripts were the most prevalent (*PtnanH1* 59% and *PtnanH2* 65% of samples) and abundant, followed by *Gardnerella* (*GnanH1* 38 to 48%, *GnanH2* 11%, *GnanH3* 19% of samples), *P. amnii* (*PananH* 25% of samples), and *P. bivia* (*PbnanH* 36% of samples), while *P. denticola* was not detected (*Pd*nanH 0%) (Fig. 5*D*). Interestingly, *Gardnerella nanH1* transcripts were more prevalent than those encoding the active *Gardnerella* sialidases (*G. piotii nanH2* and *nanH3*). Sialidase genes from additional taxa identified in Fig. 4 have low prevalence in MGs (<15% of samples) and had minimal to no presence in the MTs.

The variable prevalence of different sialidase genes and transcripts across samples raised the question of their contributions to overall sialidase expression within individual samples. To determine which sialidase genes are expressed in individual vaginal microbiomes, we computed the percent contribution of all sialidases within paired MG and MT samples (Fig. 5*E*). We found that, although *Gardnerella* sialidase genes are present in MGs, sialidase genes from *P. timonensis* and other *Prevotella* contribute a higher percentage of reads in MTs across samples from all CSTs (Fig. 5*E* and *SI Appendix*, Fig. S16*A*). *P. timonensis* sialidase transcripts were also more prevalent than all *Gardnerella* sialidases across all CSTs (*SI Appendix*, Figs. S16–S22). We also repeated the analysis, excluding the predicted inactive *Gardnerella* sialidase genes *GnanH1 and GnanH4* (*SI Appendix*, Fig. S23). This further demonstrated that *Prevotella* sialidases are the predominant genes and transcripts present across samples. Together these findings show that while both *Prevotella* and *Gardnerella* sialidase genes and transcripts are detected in vaginal samples, those from *Prevotella* are most prevalent and abundant across all CSTs. Importantly, this pattern is not simply a reflection of vaginal microbiome community composition, as previous analyses of these datasets revealed a higher abundance of *Gardnerella* (21). Altogether, our analyses indicate that *Prevotella* bacteria, particularly *P. timonensis*, are likely a predominant source of sialidase activity within the vaginal microbiome.

## Discussion

Sialidase activity in the vaginal microbiome has been strongly linked to negative health outcomes, including BV and preterm birth. Thought to significantly alter the vaginal ecosystem, this metabolic activity may facilitate mucus degradation, release carbon sources for vaginal bacteria, and promote bacterial binding to mucins and host cell glycans by revealing cryptic binding sites (36, 37). Prior to our work, knowledge of sialidase enzymes in vaginal bacteria was largely limited to *Gardnerella*. *Gardnerella* encode three sialidases, NanH1 (which is poorly active) as well as NanH2 and NanH3, which display sialidase activity and are predicted to localize extracellularly. Other vaginal bacteria were reported to possess sialidase activity in culture, including *P. bivia* and *P. timonensis* (6, 20), with a recent report implicating *P. timonensis* sialidases in vaginal cell surface glycan degradation (37). Here, we combine bioinformatic analyses and biochemical experiments to identify *Prevotella* sialidase enzymes and assess their distribution across vaginal isolates and vaginal microbiomes.

Though enzyme-encoding genes can often be annotated in bacterial genomes, important aspects of their activity cannot be readily predicted from sequence alone, highlighting the importance of biochemical characterization. Indeed, our in vitro studies of vaginal sialidases revealed differences in pH activity range, activity toward mucin substrates, and susceptibility to inhibition. Our pH analysis reveals *P. timonensis Pt*NanH1 and *Pt*NanH2 and *Gv*NanH3 are active at pH values <4.5 and >4.5, while the other *Prevotella* sialidases tested only displayed activity at pH >4.5. This finding suggests that *P. timonensis* sialidases may be active in the more acidic conditions generated by *Lactobacillus*-dominated communities. We found that only *Pt*NanH2 and GvNanH3 accept mucin substrates (MUC5B and BSM) perhaps indicating a higher potential to alter the structure and function of these important host glycans. One caveat of our work is the use of salivary MUC5B (38) rather than cervical MUC5B, which is not readily available. Though salivary and cervical MUC5B are thought to share certain glycosylation patterns, including sialylation, additional studies from more subjects are needed to elucidate the structures of cervical mucins glycans (9).

Our findings add to growing evidence that vaginal bacterial sialidases alter the structures of host glycans, likely impacting their functions. A recent study found that membrane-bound glycans of human vaginal epithelial cells from subjects with BV have diminished sialylation and recapitulated this phenotype in cells with recombinant *Gardnerella* sialidases (*Gv*NanH2 and *Gv*NanH3) (39). This finding, along with a recent report that *P. timonensis* removes sialic acids from vaginal epithelial cell surfaces (37), further implicates vaginal bacterial sialidases in detrimental host phenotypes (37).

Viral and pathogenic bacterial sialidases have been targets for small molecule inhibitor development. While the broad-spectrum inhibitor Neu5Ac2en inhibited all vaginal bacterial sialidases, the viral sialidase inhibitor Zanamivir specifically inhibited both *P. timonensis* sialidases. Though we did not observe inhibition of *Gv*NanH3 by Zanamivir in our experimental setup, it is possible that this enzyme can be partially inhibited as was previously reported (40). Segui Perez et al., demonstrate that removal of sialic acids from vaginal epithelial cell surface glycans by *P. timonensis* can be prevented by treatment with Neu5Ac2en and Zanamivir (37). Sialidase inhibitors are therefore useful tools to understand the effects of sialidase activity in the vaginal environment and could be further explored therapeutically.

Using comparative genomics, we found the sequences of *Prevotella* sialidase genes, and their presence, are more conserved across related strains compared to *Gardnerella* sialidases. Previous studies identified sialidase activity in most *P. bivia* isolates (33), but few studies have examined *P. timonensis*. Examining a diverse isolate genome collection also allowed us to find additional putative sialidases from closely related *Prevotella* species. We predict the newly identified *Gardnerella* sialidase *G*NanH4 is likely inactive, similar to *G*NanH1, since it also lacks a signal peptide and no strains encoding this gene are known to have sialidase activity. *G*NanH4 and *G*NanH1 have predicted Ig/Lectin-like domains that can also be found in *Actinomyces* sialidases, which have not been biochemically characterized. It is possible that *G*NanH1 and *G*NanH4 act intracellularly on as-yet-uncharacterized substrates given their lack of a signal peptide, or are inactive, similar to the Group B *Streptococcus* sialidase NonA (34). Future work should examine *G*NanH1 sialidase activity toward additional substrates such as human vaginal epithelial cell surface glycans (39), glycan arrays (41), or in more complex or natural settings (20, 42).

Our analyses of vaginal MGs and MTs reveal that most sialidase gene expression comes from *Prevotella* and *Gardnerella* while putative sialidase genes from other vaginal bacteria are rarely expressed. Among the *Gardnerella* sialidase genes, *GnanH1* was the most prevalent in MTs. This is perhaps surprising considering experimental evidence shows *Gv*NanH1 has extremely low activity toward sialylated substrates (13). *GnanH1* may be more prevalent in MTs because it is more frequently found in *Gardnerella* genomes compared to *GnanH2* and *GnanH3* (15); however, it may also participate in an uncharacterized metabolic process. Notably, the genes encoding *Gardnerella’s* active sialidases, *GnanH2* and *GnanH3,* were less prevalent than the *P. timonensis* sialidase genes *PtnanH1* and *PtnanH2* in this dataset, suggesting a reduced impact on host glycan metabolism. Together, these data suggest that, in addition to *Gardnerella*, *P. timonensis* makes a substantial contribution to vaginal sialidase activity.

Sialidases activity is considered a hallmark of BV^8^ and diverse CSTIV communities. Our data suggest sialidase genes and transcripts have the highest prevalence and abundance in CSTIV samples, however, we were surprised to detect sialidase gene expression in samples from other CSTs, including *L. crispatus*–dominated CSTI (*SI Appendix*, Fig. S15). The presence of sialidase genes and transcripts in other CSTs may suggest sialidases might be important in mediating transitions between CSTs. Although rare, several studies have detected sialidase activity in non-BV samples (6, 43, 44). We also noticed samples can vary in their sialidase expression profile, with some expressing three to four sialidases, while others express a single sialidase. This hints at the relevance of multiple, diverse sialidases in the vaginal environment, potentially creating a division of labor or even the potential for public good exploiters as seen in other ecosystems (45). We find that *Prevotella* sialidase transcripts are particularly prominent in MTs from all CSTs. Other studies have noted while the relative abundance of *Prevotella* species in vaginal MGs may be low (1), their genes can be highly expressed in MT data (21). In particular, the presence of *P. timonensis* sialidase transcripts in MTs from certain *Lactobacillus*-dominated communities suggests the possibility that these enzymes could contribute to community destabilization and transition to other CSTs.

Together, these bioinformatic analyses provide a more complete understanding of the origins of sialidase activity in the vaginal microbiome and highlight the importance of integrating bioinformatic analysis with detailed biochemical studies. Additional analyses of vaginal bacterial sialidase genes and transcripts in other clinical cohorts are needed, including pregnancy cohorts that could reveal links between specific sialidases and birth outcomes. A limitation of this work is that we did not biochemically characterize all the putative sialidases that were expressed in MTs to confirm their activity.

In summary, we demonstrate that *Prevotella* possess sialidase enzymes that likely play an important, currently underappreciated role in the vaginal microbiome, with *P. timonensis* standing out as a prominent source of sialidase enzymes and a major contributor of sialidase genes and transcripts in vaginal communities. A contemporary study also demonstrates that *P. timonensis* can efficiently adhere to the vaginal epithelium and that its sialidases and fucosidases are highly effective at removing glycans from the vaginal epithelial surface (37). While *Gardnerella* has been previously considered primarily responsible for sialidase activity in this environment, our work indicates that *Prevotella* species should also be included in future studies and considered in developing therapeutics targeting the vaginal microbiome. These findings highlight the need for further investigation into the biological roles of vaginal bacterial sialidases and their contributions to negative health outcomes.

## Materials and Methods

### Plasmid Construction.

Full-length sialidase genes were amplified (Q5 polymerase, New England Biolabs, M0492S) from purified genomic DNA (Qiagen, 12224-50) from vaginal bacteria (*SI Appendix*, Table S2) using primer pairs (*SI Appendix*, Table S1). The resulting gene products were assembled into pET28a expression vector (Novagen, 69864) using Gibson assembly (New England Biolabs, E2611S) and transformed into DH5α *E. coli* chemically competent cells. The identities of the constructs were confirmed by DNA sequencing (Genewiz). The confirmed plasmids were transformed into *E. coli* BL21 (DE3) for expression. All constructs were grown in LB (Research Products International, RPI, L24060-2000) containing 50 µg/mL kanamycin sulfate (VWR, 408-EU-25G).

### Sialidases Used for Expression.

The protein accession numbers in the NCBI database for each enzyme characterized are as follows: *G. vaginalis* JCP8066 NanH3 (EPI58250.1), *G. vaginalis* ATCC14018 NanH1 (WP_013399386.1), *P. bivia* DNF00188 NanH (KGF23106.1), *P. denticola* DNF00960 (WP_036854057.1), and *P. timonensi*s CRIS 5C-B1 NanH1 (WP_008123113.1) NanH2 (WP_008122213.1).

### Heterologous Expression and Purification of Heterologously Expressed Enzymes.

Chemically competent *E. coli* BL21(DE3) cells were transformed with pET28 plasmids encoding Sialidases as N-terminal His_6_-tagged fusion proteins. The transformed cells were plated onto LB_KAN_ agar plates, incubated at 37 °C overnight, and a single colony was used to grow a starter 3 mL culture and used to create a 20% glycerol stock. A 2.5 mL starter culture grown to saturation overnight was used to inoculate 1 L of LB (Research Products International, RPI) containing 50 µg/mL kanamycin. The 1 L culture was grown at 37 °C with shaking (180 rpm). When the culture reached an OD_600_ between 0.4 and 0.8, protein expression was induced by adding Isopropyl β-D-thiogalactoside (IPTG) (TEKNOVA, I3325) to a final concentration of 250 µM. The cultures were incubated at 16 °C with shaking (180 rpm) for 18 h. Cell cultures were then harvested by centrifugation at 6,000×*g* for 10 min at 4 °C and stored at −80 °C. Thawed cell pellets were resuspended in Buffer A containing 20 mM Tris, 500 mM NaCl, 10 mM MgSO_4_, 1 mM CaCl_2_, and 5 mM imidazole, and pH balanced at pH 7.5. The resuspended cells were supplemented with 0.1 mg/mL DNAse (Sigma, DN25-1G), 0.5 mg/mL Lysozyme (Sigma, L6876-10G), and Pierce Protease Inhibitor Tablets (Thermo Scientific, A32965). Cells were lysed using a cell disrupter (Emulsiflex-C3, Avestin) by passing three times at 15,000 psi, and cell lysates were clarified by centrifugation at 9,000 rpm for 45 min at 4 °C. The clarified lysate was transferred to a 15 mL column containing 3 mL Ni-NTA Agarose affinity resin equilibrated with buffer A at 4 °C (Invitrogen). The column was washed using a gradient of Buffer A containing 20 mM, 50 mM, 75 mM, and 100 mM imidazole. Protein was eluted with buffer A containing 250 mM imidazole. Fractions were analyzed by SDS-PAGE (Biorad, 4561086) and stained with InstantBlue Coomassie protein stain (Abcam, ab119211). The protein-containing fractions were pooled and concentrated in an Amicon spin concentrator of 10 to 30 kDa cutoff (Millipore, UCF901024 and UCF903024) and buffer exchanged against Buffer B (20 mM Tris, 100 mM NaCl, 10 mM MgSO_4_, 1 mM CaCl_2_, 10 % glycerol, pH 7.5). Protein aliquots of 20 µL were frozen in liquid nitrogen and stored at –80 °C. Protein concentrations were estimated with a NanoDrop 2000 UV–Vis Spectrophotometer (Thermo Scientific) using the theoretical molar absorption coefficient calculated using https://web.expasy.org/protparam/.

### Sialidase Activity Screen in Vaginal Isolates.

Strains were grown anaerobically inside an anaerobic chamber with an atmosphere of 2.5 % H_2_, 5 % CO_2_, 92.5 % N_2_ (Coy Lab Products) in 96-well plates containing 200 µL of PYGT media (1 % glucose) supplemented with 10 % horse serum broth for 48 h. Cultures were then normalized to OD = 1 and 20 µL of culture was added to a black 96-well plate containing 350 µM 4-MU-Neu5Ac (Biosynth) dissolved in 80 µL of sodium acetate buffer pH 5.5. Fluorescence signal was monitored at 360/440 nm for 105 min in a plate reader (Biotek Synergy HTX) at 37 °C.

### Activity Assays of Purified Sialidases.

Sialidase activity was measured using a fluorescence-based assay in a total volume of 100 µL, prepared in a black, 96-well polystyrene plate (Corning). Each well contained 2.5 nM purified enzyme in sodium acetate buffer (100 µM, pH 5.5) and 4-MU-Neu5Ac (200 µM) was added to start the sialidase reaction. Fluorescence 330/440 nm was measured while incubating at 37 °C with shaking in a plate reader (Biotek).

### Kinetic Characterization.

Sialidase activity was measured using 2.5 nM purified enzyme in sodium acetate buffer (100 µM, pH 5.5) in a total volume of 100 µL. 4-MU-Neu5Ac was diluted to create a 12-step, 1:2 dilution series from 800 to 3.1 µM. Fluorescence 360/440 nm was measured while incubating at 37 °C with shaking in a plate reader (Biotek). Slopes were calculated over the first 5 min, and GraphPad was used to calculate Michaelis–Menten kinetic parameters, V_max_ and K_m_.

### pH Profiles of Sialidases.

The optimal pH for enzyme activity was determined by incubating 20 to 100 nM sialidases with 200 µM 4-MU-Neu5Ac in Mcllvaine buffer (0.1 M citric acid/0.2 M phosphate buffer) at a pH range of 3 to 8 for 30 min in at 37 °C. Activity was assayed by monitoring fluorescence 360/440 nm in a plate reader (Biotek). The assay was prepared in a 384-well, black, flat-bottom polystyrene plate (Corning). Fluorescence values were normalized to standard curves of 4-methylumbelliferone (Sigma) (concentration 200 to 1.5 µM) dissolved in DMSO and prepared in the same citrate/phosphate buffers from pH 3 to 8. Relative sialidase activity was determined by measuring the total concentration of 4-methylumbelliferone released at 29 min.

### Sialidase Substrate Specificity Endpoint Assay.

To determine the substrate specificity of sialidases, 40 nM enzyme was incubated with the following substrates: 4-MU-Neu5Ac (250 µM), BSM (1 mg/mL), human serum IgA (0.5 mg/mL), human serum IgG (4 mg/mL), 3′sialyllactose (200 µM), 6′siallylactose (200 µM), MUC5B (0.5 mg/mL) in 20 mM sodium acetate buffer pH 5.5 in a total volume of 50 µL. All samples included *N*-acetyl-D-neuraminic acid-1,2,3-^13^C_3_ (Sigma, 649694) as an internal standard. The assays were incubated for 2 h at 37 °C in a thermocycler (Biorad). Samples were then filtered through 3 kDa filter plates and derivatized with 4,5-dimethoxy-1,2-phenylenediamine hydrochloride (DMB), (Sigma, 36271). To derivatize samples, 20 µL of filtered sample was mixed with 80 µL of derivatization reagent (6.5 mM DMB, 52 mM sodium dithionite, 0.75 M 2-mercaptoethanol, 1.33 M acetic acid). Samples were incubated at 50 °C for 2 h.

### Anaerobic Culture Conditions.

All plastic consumables used for culturing were kept in the anaerobic chamber for at least 24 h prior to the experiment. Bacterial isolates were obtained from Biodefense and Emerging Infections Research Resources Repository (BEI Resources) *SI Appendix*, Table S2). *Gardnerella* and *Prevotella* strains were cultured in PYGT media as previously described (46) (20 g peptone, 10 g glucose, 10 g yeast extract, 0.4 g sodium bicarbonate, 40 mg dipotassium phosphate, 40 mg monopotassium phosphate, 5 mg hemin, 5 mg vitamin K, 0.25 g L-cysteine hydrochloride, 8 mg magnesium sulfate, 0.250 mL TWEEN-80, 50 mL heat-inactivated horse serum (Sigma), in 1 L of filter-sterilized water (0.2 µm), containing 10 % inactivated horse serum. Medium was transferred to an anaerobic chamber with an atmosphere containing 2.5 % H_2_, 5 % CO_2_ and 92.5 % N_2_ (Coy Labs). Starter cultures of *Gardnerella* and *Prevotella* were inoculated in 96-well plates containing 200 µL of PYGT and incubated at 37 °C overnight.

### Sialic Acid Detection by DMB Labeling and LC–MS Analysis.

Derivatized samples were prepared for analysis by diluting 1:100 in 90:10 acetonitrile (ACN): water. See derivatization procedure in *SI Appendix*, Supplemental Methods. Samples were analyzed by ultra-high-performance model Xevo TQ-S (Waters, UPLC-MS/MS), see *SI Appendix*, Supplemental Methods and Materials (*SI Appendix*, Table S4).

### MUC5B Purification.

Submandibular saliva was collected in bulk from human volunteers using a custom vacuum pump, as described previously (38, 47). We collected the saliva samples after explaining the nature and possible consequences of the studies, obtaining written informed consent, and receiving approval from the institutional review board and Massachusetts Institute of Technology (MIT)’s Committee on the Use of Humans as Experimental Subjects under protocol #1312006096. Immediately after collection, salts, antibacterial agents, and protease inhibitors were added to the saliva to reach a final concentration of 0.16 M NaCl, 5 mM benzamidine HCl, 1 mM dibromoacetophenone, 1 mM phenylmethylsulfonyl fluoride, and 5 mM EDTA. The mucins in the saliva were solubilized overnight by stirring gently at 4 °C. Solubilized saliva was then flash-cooled in liquid nitrogen in 10 to 40 mL volumes and stored at –80 °C. Before chromatography, 200 mL of saliva from separate donors was thawed at 4 °C, and insoluble material was removed by centrifugation at 10,000×*g* for 10 min at 4 °C. MUC5B was purified using a Bio-Rad NGC fast protein liquid chromatography (FPLC) system equipped with an XK 50 column packed with 2 L of Sepharose CL-2B resin (GE Healthcare Bio-Sciences). Mucin-containing fractions were identified using a periodic acid–Schiff's reagent assay and analysis of UV absorbance at 280 nm from FPLC. Fractions were then combined, dialyzed, and concentrated using an ultrafiltration device and were then lyophilized for storage at −80 °C. Protocols involving the use of human subjects were approved by Massachusetts Institute of Technology’s Committee on the Use of Humans as Experimental Subjects.

### Inhibition of Purified Sialidases.

Sialidase inhibition assays were performed using 5 nM purified enzyme in sodium acetate buffer (100 µM, pH 5.5) and with varying concentrations of Neu5ac2en (Sigma, D9050) in a total volume of 50 µL. Each well contained 1 µL of enzyme was premixed with 39 µL of buffer and 5 µL of inhibitor in a black 384-well flat-bottom polystyrene plate (Corning) and 5 µL of 4-MU-Neu5Ac (100 µM final concentration) was added to start the assay. The plate was immediately transferred into the plate reader to incubate at 37 °C with shaking to measure fluorescence 360/440 nm. For improved accuracy, the enzyme, inhibitor, and 4-MU-Neu5Ac were dispensed into the plate using a Formulatrix MANTIS. Slopes to determine sialidase activity were calculated over the first 5 min, and the IC_50_ were determined by nonlinear fit [Inhibitor] vs. response on GraphPad Prism.

### Bacterial Genomic Database and HMM-Based Sialidase Searches of Isolate Genomes.

A bacterial isolate genomic database was constructed using 1,189 published genomes from the Vaginal Microbiome Research Consortia, spanning cohorts from the United States and South Africa (Full list and metadata provided in Supplementary data file). Bacterial genomes were organized by phylogeny (*SI Appendix*, Figs. S10, S11, S13, and S15) using a concatenated ribosomal protein tree. We used HMMER (v3.3.2) to find ribosomal proteins, aligned the sequences with MAFFT (v7.508) and used RAxML (v.8.2.10) to create the phylogenetic trees. In order to search our diverse collection of vaginal bacterial genomes for putative sialidases, we used HMMER (v3.3.2) to construct a hidden Markov model using a database made of a multiple sequence alignment of nine sialidase genes (See supplementary fasta file A) constructed with MAFFT (v7.508). We required all hits to be greater than 250 amino acids in length (*SI Appendix*, Supplementary Fasta File B) and validated hits manually by searching for predicted sialidase domains using InterProScan.

### MG and MT Searches and Quantification.

Diamond blastX was used to quantify the abundance of sialidase encoding genes and transcripts in MG and MT published by the Vaginal Microbiome Research Consortium (VMRC). First, we compiled all the candidate sialidase genes identified by HMMER and created representative sequences using CD-hit with >85 % amino acid ID. After representative sequences were generated, they were used in Diamond blastX with a e-value cutoff of <e-20 and a percent amino acid sequence identity >50 % to determine the abundance of these sialidases in MG and MT databases generated by the VMRC under the Bioproject PRJNA797778. By setting a stringent identity and e value cutoff, we are quantifying high confidence hits to specific sialidase genes queried. The scripts for processing the datasets were described previously (21). We analyzed 176 paired MG and MT from 40 patients. The output from diamond blastx is RPKM. Sample metadata was used to bin the results by community state type (CSTI n = 39, CSTII n = 16, CSTIII n = 31, CSTIV n = 80, CSTV n = 10). The total expression per sample was calculated by summing all the RPKM values for all the candidate sialidases for each sample. Prevalence is defined by detecting any sialidase read in a sample. Percent contribution of a sialidase is determined by the relative abundance of one sialidase divided by the sum of all sialidase abundances within each sample. Plots were generated using Python 3 and PRISM.

## Supplementary Material

Appendix 01 (PDF)

Dataset S01 (XLSX)

## Data Availability

The bacterial isolate genomes are publicly available under the BioProject accession codes: PRJNA799634 (48), PRJNA799744 (49), PRJNA799737 (50), PRJNA799746 (51), and PRJNA797778 (52). Experimental data have been deposited in Synapse.org (https://doi.org/10.7303/syn53254095) (53). Previously published data were used for this work (21).
