# Supplementary material for: Prevotella are major contributors of sialidases in the human vaginal microbiome
Source: Proc Natl Acad Sci U S A. 2024 Aug 26;121(36):e2400341121. doi: 10.1073/pnas.2400341121 (PMC11388281; doi:10.1073/pnas.2400341121)
Supplement: Supplementary file 1 — Appendix 01 (PDF) [file pnas.2400341121.sapp.pdf]

PNAS

Supporting Information for
Prevotella are major contributors of sialidases in the human vaginal microbiome.

Paula Pelayo[1], Fatima A. Hussain[2], Caroline A. Werlang[3], Chloe Wu[3], Benjamin M. Woolston[1,4], Claire M. Xiang[1], Lindsay Rutt[5], Michael T. France[5], Jacques Ravel[5], Katharina Ribbeck[3], Douglas S. Kwon[2], Emily P. Balskus[1,6].

Emily P. Balskus.
Email: balskus@chemistry.harvard.edu

This PDF file includes:

Supplemental Methods and Materials
Figures S1 to S23
Tables S1 to S8
Supplemental References

Other supporting materials for this manuscript include the following:

Supplementary data file.xls
Supplementary fasta file A.faa
Supplementary fasta file B.faa

Supplemental Methods and Materials

All chemicals and solvents were purchased from Sigma-Aldrich, except where otherwise noted.

Table S1. Primers used for cloning sialidases.

Name	Description	Sequence (5' to 3')
oPP_01	pET28a-F (N-His + Thrombin)	GCTGCCGCGCGGCACCAG
oPP_02	pET28a-R (Eliminate other tags)	CAAAGCCCGAAAGGAAGCTG
oPP_03	P. bivia DNF00188 sialidase-F	cacagcagcggcctggtgccgcgcggcagcCAAGATACCTTGTTTGTTAAAC
oPP_04	P. bivia DNF00188 sialidase-R	gcagccaactcagcttcctttcgggctttgTTATTTCTCTTTGATAATATCT
oPP_05	P. denticola DNF00960 sialidase-F	cacagcagcggcctggtgccgcgcggcagcGCCGGGACAGACAGGAACAGAG
oPP_06	P. denticola DNF00960 sialidase-R	gcagccaactcagcttcctttcgggctttgTCAGCGGCGACGTGCGGCAGCG
oPP_07	P. timonensis cris 5c-b1 sialidase long-F	cacagcagcggcctggtgccgcgcggcagcGCGGACAAGGTAATCCGCATTC
oPP_08	P. timonensis cris 5c-b1 sialidase long-R	gcagccaactcagcttcctttcgggctttgTTACTTCACCACCACCTTTTTG
oPP_09	P. timonensis cris 5c-b1 sialidase short-F	cacagcagcggcctggtgccgcgcggcagcTCAAACAACCAGCATCACCAAC
oPP_10	P. timonensis cris 5c-b1 sialidase short-R	gcagccaactcagcttcctttcgggctttgTTAGTAGCCCTTCTTGAAGCGT
oBMW_011	G. vaginalis JCP8066 NanH3-F	cagcggcctggtgccgcgcggcagcACTACCCCCCCCATGAAC
oBMW_012	G. vaginalis JCP8066 NanH3-R	caactcagcttcctttcgggctttgTTAATATTTCATATTTTTTAATTTCATTAATGCTGTAGAGATAGCTATTAATAGAAG
oBMW_013	G. vaginalis ATCC14018 NanH1-F	cagcggcctggtgccgcgcggcagcATGGAACGTCGTTCAACG
oBMW_014	G. vaginalis ATCC14018 NanH1-R	caactcagcttcctttcgggctttgTTAAATGTCTCTTCCATGTTGG

Table S2. Bacterial strains used for sialidase characterization and assays of sialidase activity.

Sialidase	Cloned from this strain	GenBank assembly Accession	Source
*Gv*NanH3	*G. vaginalis JCP8066*	GCF_000414565.1	Biodefense and Emerging Infections Research Resources Repository (BEI Resources)
*Gv*NanH1	*G. vaginalis ATCC14018*	GCA_003397685.1	BEI Resources
*Pb*NanH	*P. bivia DNF00188*	GCA_000759045.1	BEI Resources
*Pd*NanH	*P. denticola DNF00960*	GCA_000759205.1	BEI Resources
*Pt*NanH1	*P. timonensis cris 5c-b1*	GCA_000177055.1	BEI Resources
*Pt*NanH2	*P. timonensis cris 5c-b1*	GCA_000177055.1	BEI Resources

Table S3. Cloning strains (A) and (B) plasmids used in this study.

A. *E. coli* strains

Name	Description / Use	Source
E. coli DH5α	Cloning strain	NEB (New England BioLabs)
E. coli BLL21(DE3)	Protein expression and purification	NEB

B. Plasmids

Plasmid	Description	Source
pET28a-*GvnanH1*	N-$His_{6,}$ thrombin, *G. vaginalis nanH1*, Km^r	This study
pET28a-*GvnanH3*	N-$His_{6,}$ thrombin, *G. vaginalis nanH3*, Km^r	This study
pET28a-*PbnanH*	N-$His_{6,}$ thrombin, *P. bivia nanH*, Km^r	This study
pET28a-*PdnanH*	N-$His_{6,}$ thrombin, *P. denticola nanH*, Km^r	This study
pET28a-*PtnanH1*	N-$His_{6,}$ thrombin, *P. timonensis nanH1*, Km^r	This study
pET28a-*PtnanH2*	N-$His_{6,}$ thrombin, *P. timonensis nanH2*, Km^r	This study

UPLC-MS/MS methods for detecting derivatized sialic acids

Derivatized samples were prepared for analysis by diluting derivatized samples by 1:100 in 90:10 acetonitrile (ACN): water. Samples were analyzed by ultra-high performance liquid chromatography tandem mass spectrometry (UPLC-MS/MS). Simultaneous analysis of Neu5Ac, *N*-Acetyl-D-neuraminic acid-1,2,3-$^{13}C_3$ (Sigma, 649694), and Neu5Gc derivatized with 4,5-dimethoxy-1,2-phenylenediamine hydrochloride (DMB) was carried out by UPLC-MS/MS. Liquid chromatography was conducted using a Waters Aquity H-Class System (Waters Corporation). Following sample preparation, 1 µL of sample was injected onto a BEH Amide 1.7 µm (2.1 x 50 mm) column (Acquity, 186004801). The flow rate was 0.650 mL min^{-1} using mobile phase A = 0.1 % formic acid in H_2O and mobile phase B = 0.1 % formic acid in acetonitrile (ACN). The column temperature was maintained at 40 °C. The following gradient was applied: 0–1.5 min at 90-60% B, 1.5-2 min at 60% B isocratic, 2.0-2.2 min at 60-90% B, 2.2–3.5 min at 90% B isocratic. MS detection was performed with a Waters Xevo TQ-S (Waters Corporation) instrument with electron spray ionization in positive mode (ESI+) (capillary voltage,3.3 kV; cone voltage, 48 V; source offset voltage, 50 V; desolvation temperature, 450 °C; desolvation gas flow, 250 L h–1; cone gas flow, 150 L h–1; nebulizer, 4.0 bar). For quantification, standard curves for Neu5Ac and Neu5Gc ranging from 0.1–200 µM were prepared in triplicate. Standards were prepared and derivatized in parallel with experimental samples for each experiment.

Table S4. UPLC-MS/MS analysis of sialic acids used in assays.

Standard	*Transition (m/z)*	*Mode*	*Cone (V)*	*Collision (V)*	*Retention (min)*
DMB-Neu5Ac	442.33>424.298	ESI	48 V	10	1.57
DMB-^{13}CNeu5Ac	445.33>427.298	ESI	48 V	10	1.55
DMB-Neu5Gc	458.17> 440.17	ESI	48 V	10	1.67

Inhibition of sialidase activity in bacterial culture

Bacteria were grown anaerobically in PYGT media for 48 hours, diluted to an OD=1 and then 10 µL of culture was added to a black 384-well flat bottom polystyrene plate (Corning) containing sodium acetate buffer (100 µM, pH 5.5) and with varying concentrations of Neu5ac2en (Sigma, D9050) in a total volume of 50 µL. To start the assay, 5 µL of 4-MU-Neu5Ac (100 µM final concentration) was added and the plate was immediately transferred into the plate reader to incubate at 37 °C with shaking to measure fluorescence 360/440 nm for 2 hours. For improved accuracy, the 4-MU-Neu5Ac substrate was dispensed into the plate using a Formulatrix MANTIS. Slopes to determine sialidase activity were calculated over 20 points across the 2 hours and the EC_{50} were determined by non-linear fit [Inhibitor] vs. response on Graphpad Prism.

Supplemental Figures

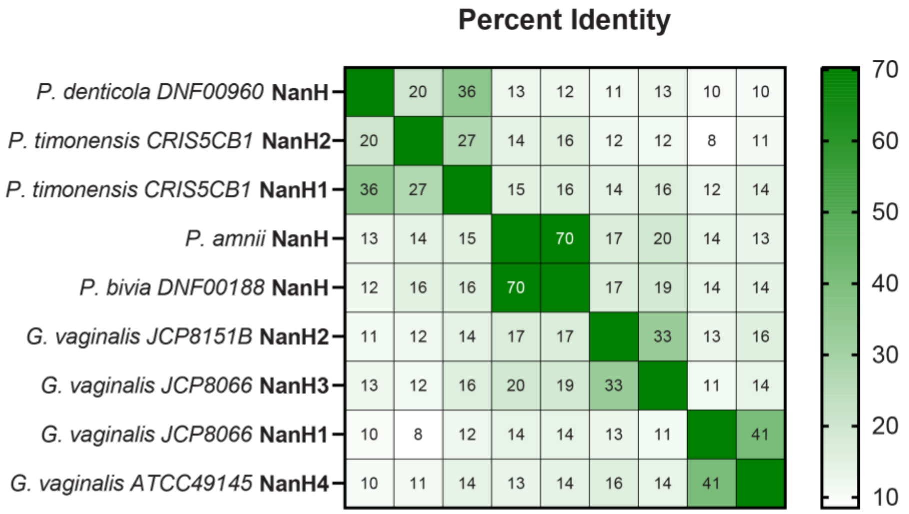

Figure S1. Muscle alignment of the full amino acid sequences from 4 *Gardnerella* and 5 *Prevotella* sialidases.

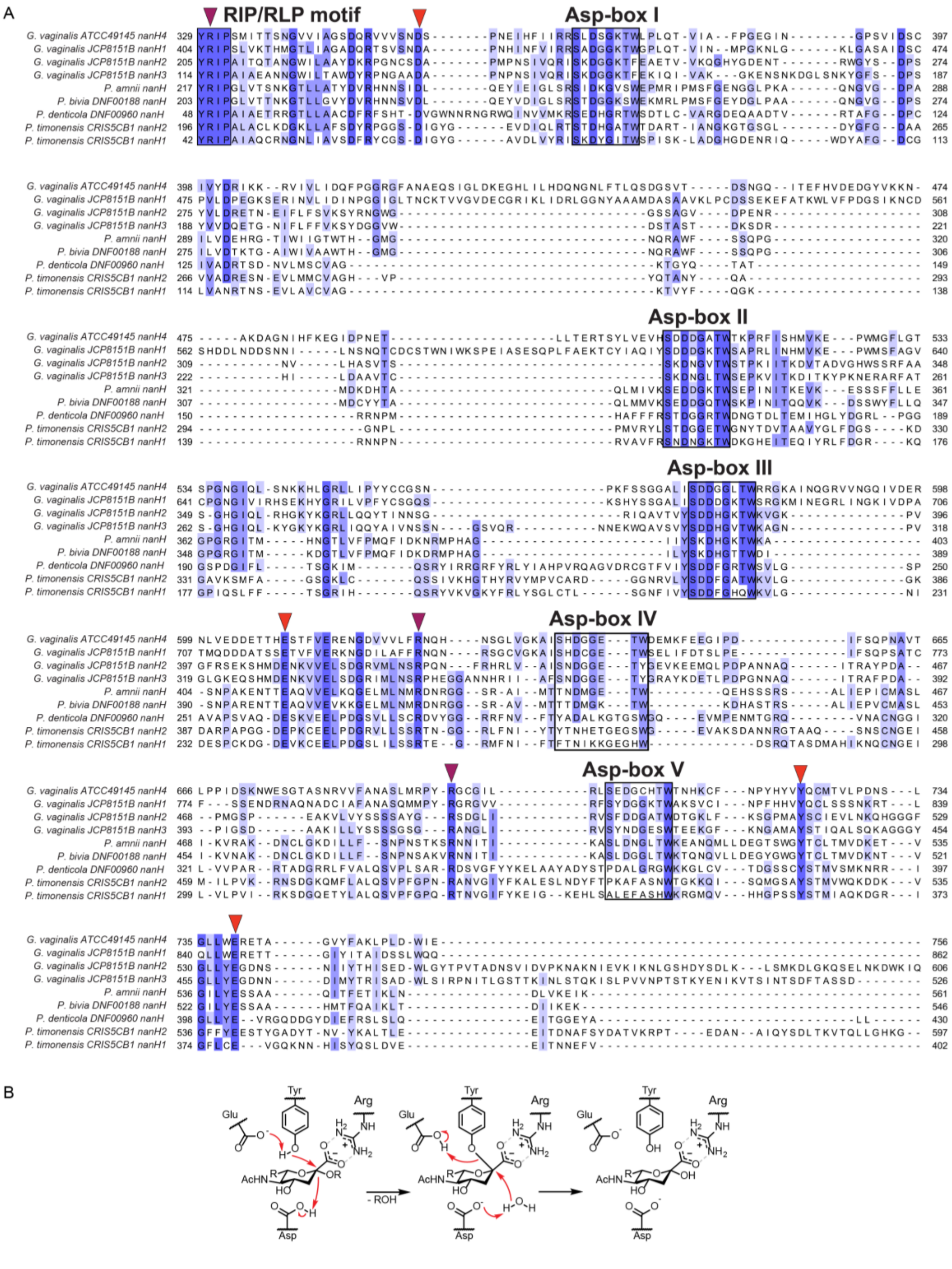

Figure S2. Multiple sequence alignment of 4 *Gardnerella* and 5 *Prevotella* sialidases showing the conserved active site residues involved in catalysis (red and maroon arrows). **A)** Rectangles indicate the conserved sequence motifs, RIP/RLP motif (Arg-Ile/Leu-Pro) and the signature bacterial neuraminidase repeat, BNR repeat (Ser/Thr-X-Asp-X-Gly-X-Thr-Trp/Phe). BNR repeats are also known as Asp-box motifs[1]. **B)** Proposed mechanism for sialidases.

Table S5. Active site residues conserved among *Gardnerella* and *Prevotella* sialidases.

Organism	Sialidase	Arg triad	Tyr/Glu-E	Asp-D	Glu
G. vaginalis	NanH1	405/733/800	828/717	430	844
G. vaginalis	NanH2	206/423/487	515/407	231	534
G. vaginalis	NanH3	115/345/412	440/329	140	459
G. vaginalis	NanH4	330/695/	723/609	355	732
P. bivia	NanH	204/415/479	510/399	229	526
P. timonensis	NanH1	43/257/325	362/241	67	378
P. timonensis	NanH2	197/412/485	524/396	221	540
P. denticola	NanH	49/276/347	386/260	73	402

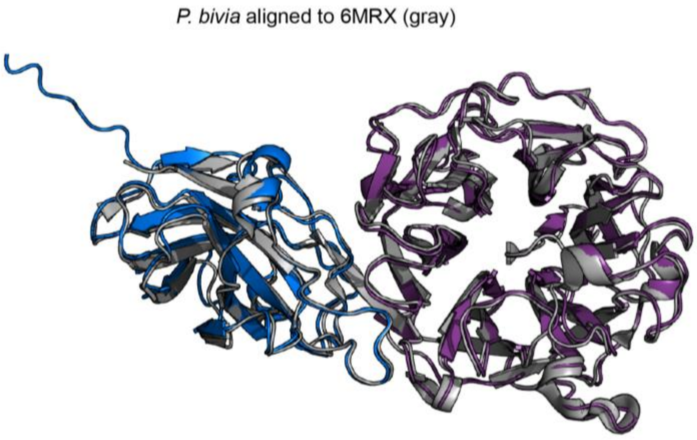

Figure S3. Predicted structures of *P. bivia* sialidase PbNanH generated via ColabFold AlphaFold2. **A)** Sialidase domain IPR01140 (purple) is present, forming the catalytic β-propeller fold domain. PbNanH contains an additional predicted sialidase carbohydrate binding domain (blue). PbNanH shares 66% amino acid ID with Sialidase26 (6MRX), aligned in gray[2].

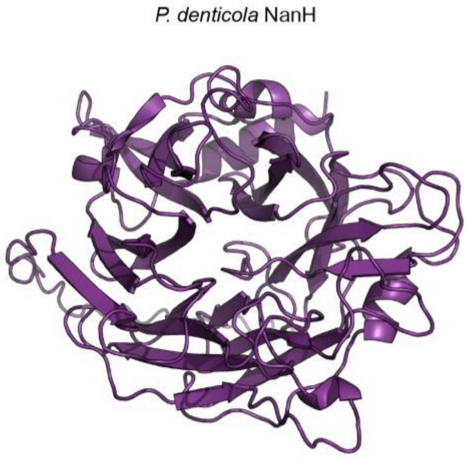

Figure S4. Predicted structures of *P. denticola* sialidase PdNanH generated via ColabFold AlphaFold2. Sialidase domain IPR01140 is present, forming the catalytic β-propeller fold domain.

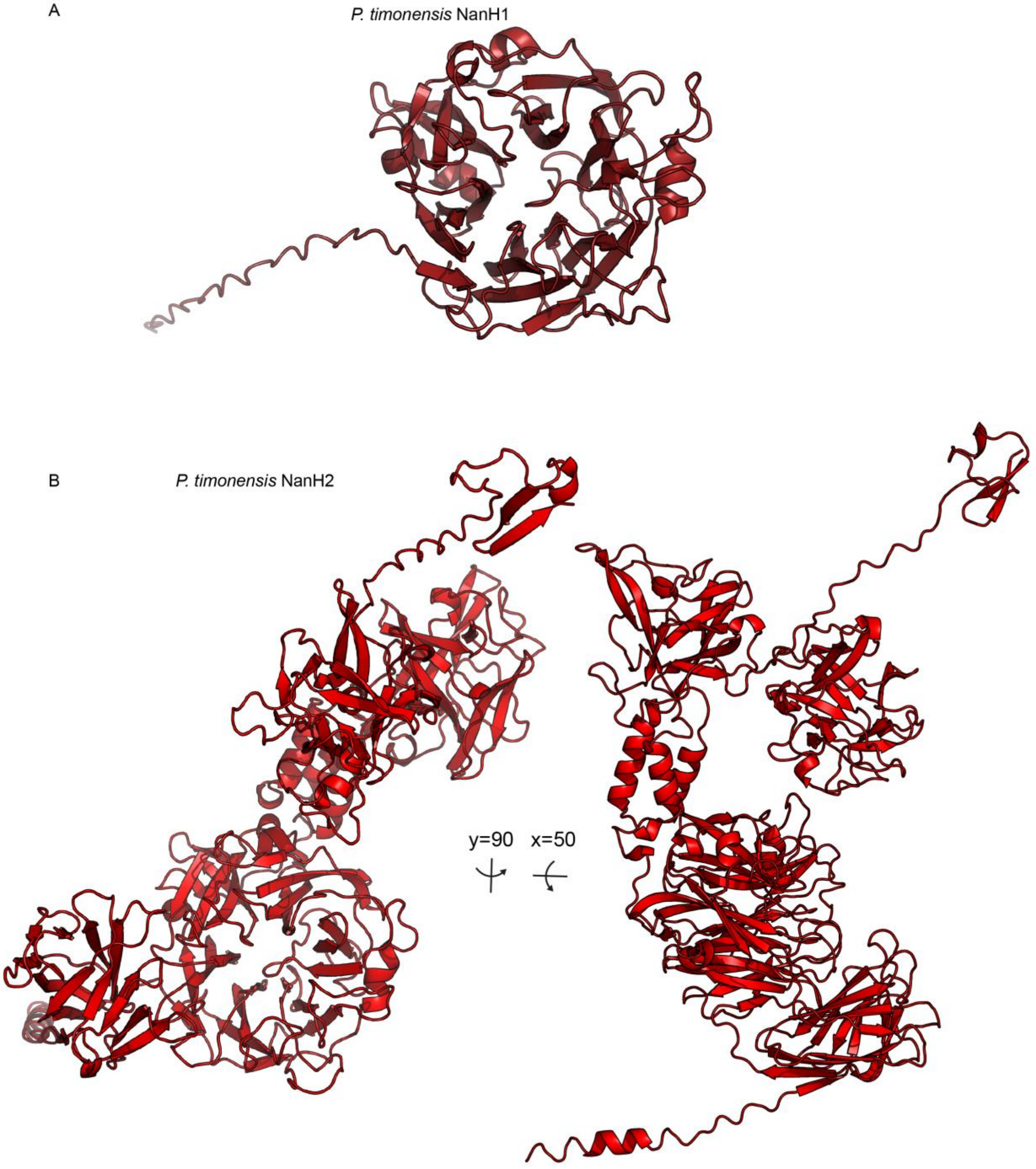

Figure S5. Predicted structures of *P. timonensis* sialidases generated via ColabFold AlphaFold2 **A)** PtNanH1 and **B)** PtNanH2. PtNanH2 contains the IPR01140 domain and 4 additional structural elements of unknown function.

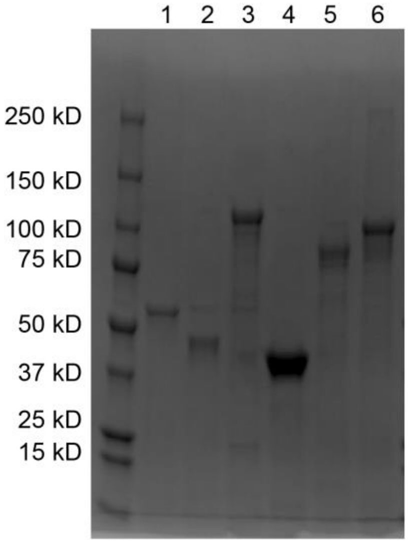

Figure S6. SDS-PAGE of recombinant purified sialidases. From left to right, Precision Plus Protein All Blue Standards (BioRad), (1) *P. bivia* NanH, 60.7 kD; (2) *P. denticola* NanH, 49 kD; (3) *P. timonensis* NanH2, 111 kD; (4) *P. timonensis* NanH1, 46 kD; (5) *G. vaginalis* NanH3, 79 kD; (6) *G. vaginalis* NanH1, 101 kD. Gel was stained with Instant Blue (Abcam).

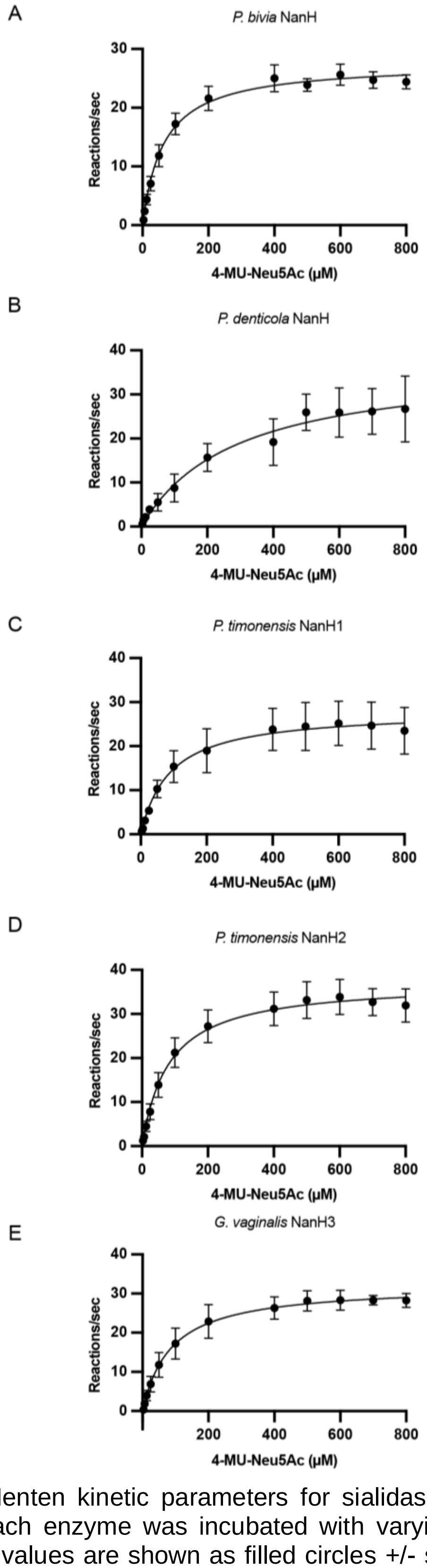

Figure S7. Michaelis–Menten kinetic parameters for sialidases. A) Kinetic characterization of purified sialidases. 2.5 nM of each enzyme was incubated with varying concentrations of 4-MU-Neu5Ac for 5 minutes at 37 °C. Mean values are shown as filled circles +/- standard error of means (SEM) from three independent experiments (n=3) using the same batch of purified enzymes on the same day.

Table S6. Kinetic parameters of sialidases for the hydrolysis of 4-MU-Neu5Ac. Values represent mean of three independent experiments on the same day (n=3). Range represents ± SD.

Enzyme	k_{cat} (s^{-1})	Catalytic efficiency k_{cat} / K_m (s^{-1} M^{-1})	K_m (μM)	V_{max} (μM min^{-1})
P. timonensis NanH1	112.20 ± 8.29	(1.26 ± 0.24) x 10^6	88.90 ± 27.14	16.83 ± 1.24
P. timonensis NanH2	149.20 ± 6.28	(1.83 ± 0.40) x 10^6	81.37 ± 14.53	22.38 ± 0.94
P. bivia NanH	110.46 ± 2.87	(1.73 ± 0.25) x 10^6	64.01 ± 7.48	16.57 ± 0.43
P. denticola NanH	149.33 ± 22.21	(0.51 ± 0.28) x 10^6	290.30 ± 113.43	22.40 ± 3.33
G. vaginalis NanH3	128.60 ± 5.38	(1.48 ± 0.32) x 10^6	86.79 ± 15.10	19.29 ± 0.81

Table S7. For comparison, kinetic parameters from previously characterized sialidases for the hydrolysis of *p*-nitrophenyl-*N*-acetylneuraminic acid (*p*-NP-Neu5Ac).

Organism	Enzyme	k_{cat} (s^{-1})	Catalytic efficiency k_{cat} / K_m (s^{-1} M^{-1})	K_m (μM)	Substrate	Study
Streptoccoccus pneumoniae	NanA	>175	(3.5 ± 0.3) x 10^5	>500	*p*-NP-Neu5Ac	3
Streptoccoccus pneumoniae	NanB	>0.14	(2.7 ± 0.3) x 10^2	>500	*p*-NP-Neu5Ac	3
Streptoccoccus pneumoniae	NanC	>17	(3.4 ± 0.3) x 10^4	>500	*p*-NP-Neu5Ac	3

Table S8. Activity of sialidase inhibitors (up to 2 mM) towards purified vaginal sialidases using 4-MU-Neu5Ac as a substrate. ND = Not determined.

	IC_{50} (μM)	
	Neu5Ac2en	Zanamivir
P. bivia NanH	103.3 ± 20.3	ND
P. denticola NanH	50.5 ± 22.5	ND
P. timonensis NanH1	180.0 ± 92.3	4426.0 ± ND
P. timonensis NanH2	56.0 ± 23.0	33.6 ± 15.5
G. vaginalis NanH3	29.0 ± 5.6	ND

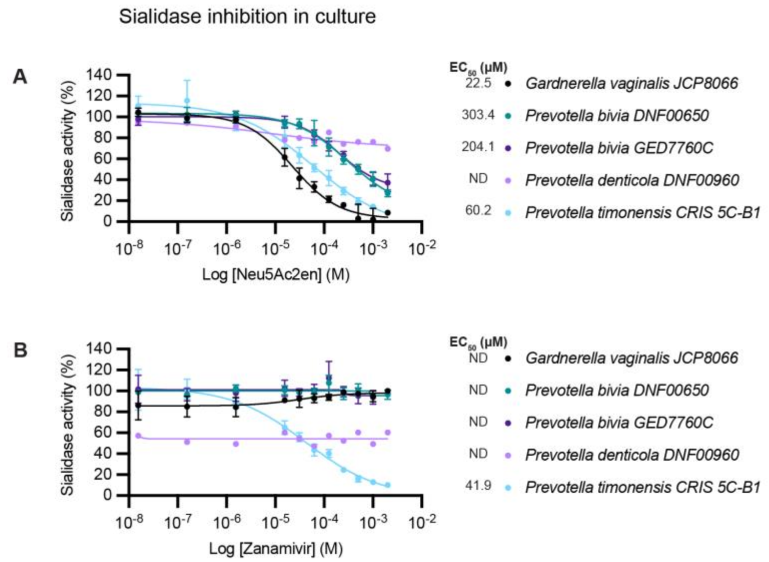

Figure S8. Inhibition of sialidase activity in *Prevotella* and *Gardnerella* cultures using 4-MU-Neu5Ac as a substrate. Inhibition activity of (A) Neu5Ac2en and (B) Zanamivir. Bacteria grown in PYGT media for 48 hours were incubated with varying concentrations of Neu5ac2en and Zanamivir for 2 h at 37 °C. Data represent the mean of 3 biological replicates ± SEM (except *P. denticola* which only has one replicate).

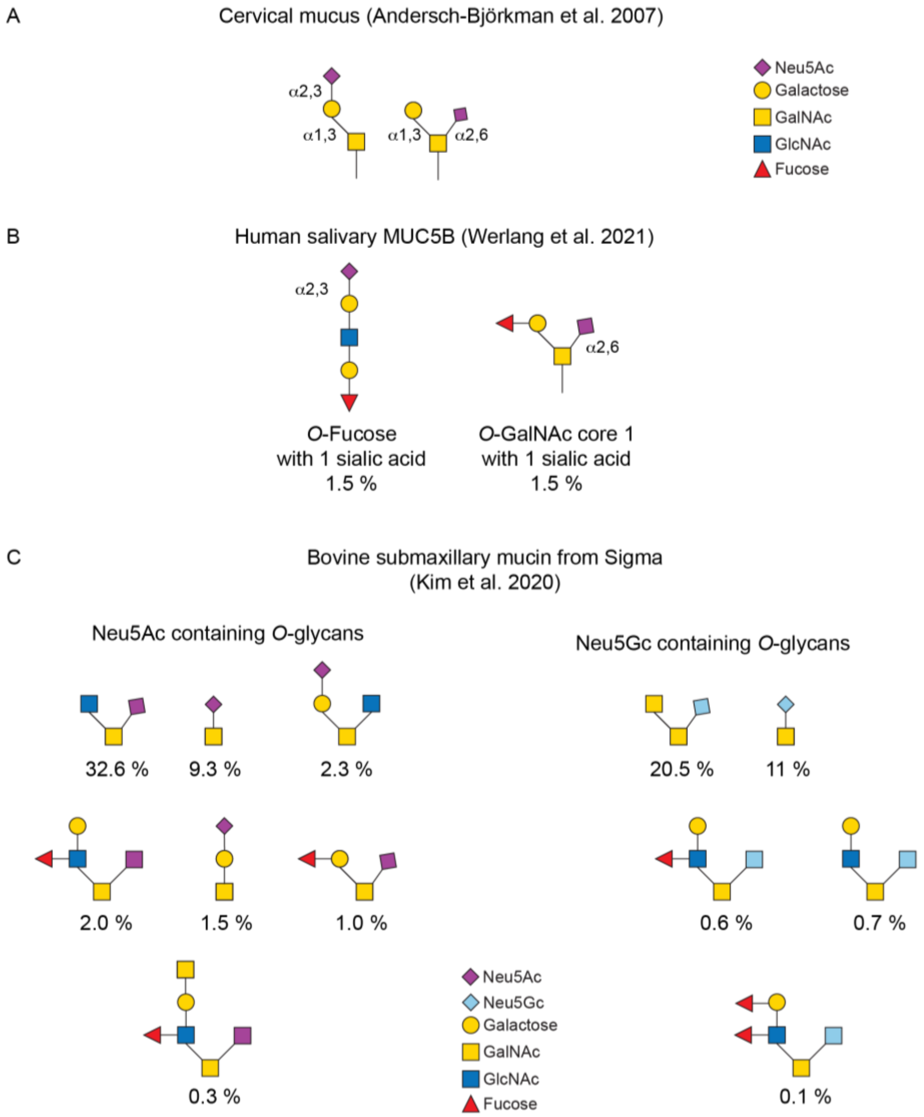

Figure S9. Glycans isolated from various mucins.
Salivary MUC5B and Bovine submaxillary mucin share certain sialylated glycan patterns with cervical MUC5B. (A) MUC5B is the major mucin found in cervical mucus[4,5]. One study characterized the *O*-glycosylation pattern of cervical mucus from 12 subjects using HPLC-MS/MS[4]. They found MUC5B was the most abundant mucin and determined mucin glycans collected before and after ovulation consisted of mainly sialylated glycans (Neu5Ac α(2,6)-GalNAc and Neu5Ac α(2,3)-Gal-GalNAc), which peak right before ovulation[4]. (B) The salivary MUC5B used in this study was analyzed in a previous publication using nanoelectrospray ionization MS with an orbital trap mass analyzer[6]. Werlang et al determined that MUC5B contains 1.5 % *O*-GalNAc core 1 with 1 sialic acid and 1.5 % *O*-Fucose with 1 sialic acid. The *O*-GalNAc core 1 with 1 sialic acid bound in the α2,6 configuration is similar to the sialic acid containing *O*-glycans found in cervical mucus. (C) Bovine submaxillary mucin (BSM) used in this study was purchased from Sigma. The sialic acid containing glycans in commercial BSM from the same source were recently described[7] highlighting various sialic acid (Neu5Ac and Neu5Gc) containing glycans. Notably, BSM contains α(2,3) Neu5Ac bound to galactose and α(2,6) Neu5Ac bound to GalNAc, similar to the cervical MUC5B mucins.

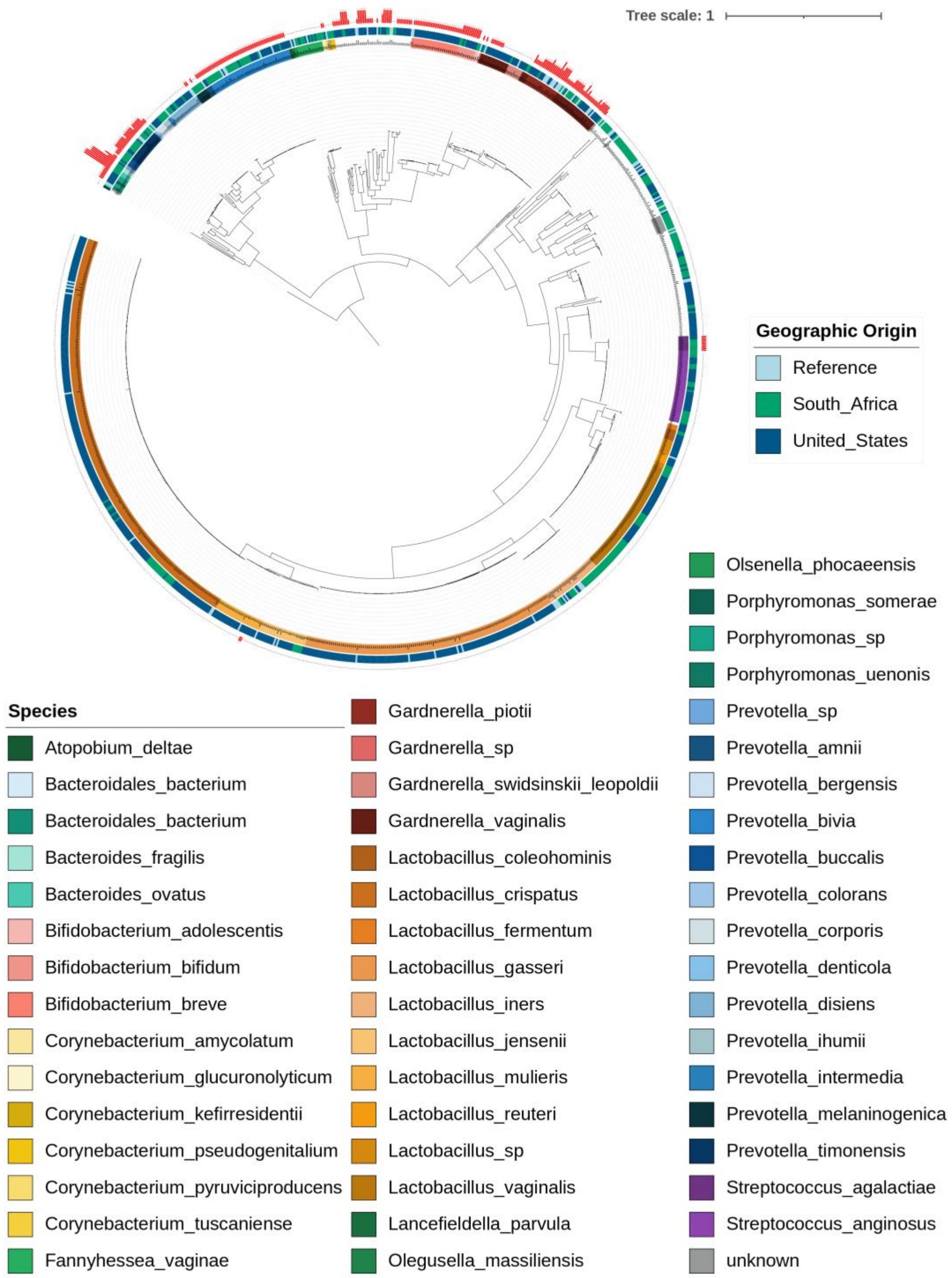

Figure S10. Sialidases encoded in vaginal isolate genomes.
Phylogenetic tree of vaginal isolates from the Vaginal Microbiome Research Consortium (N. American) and FRESH (S. African) studies. The phylogeny is based on 49 concatenated ribosomal proteins and serves as a proxy for the core genome. Each genome was searched using the sialidase protein alignment with HMMER (version 3.1b2). The scale bar indicates nucleotides substitutions per site. The presence of sialidases is represented by the red bar next to the isolate ID with the height and number indicating the number of sialidase hits.

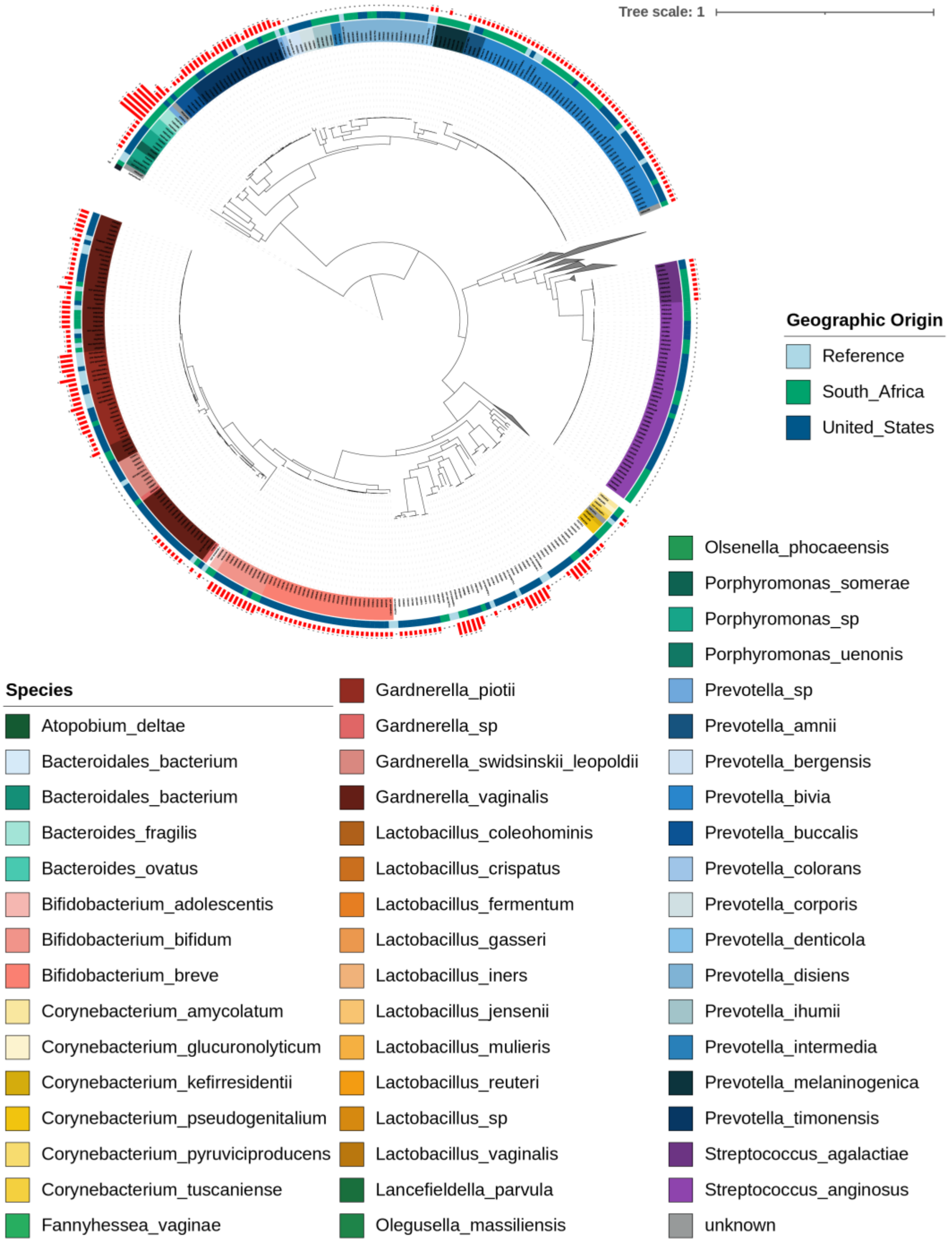

Figure S11. Sialidases encoded in vaginal isolate genomes with *Lactobacillus* clade collapsed
This figure is identical to Figure S10, but with the *Lactobacillus* clade collapsed. Sialidases are shown in red bars next to the isolate ID and the height indicates the number of sialidase hits. Phylogenetic tree of vaginal isolates from the Vaginal Microbiome Research Consortium (N. American) and FRESH (S. African) studies. The phylogeny is based on 49 concatenated ribosomal proteins and serves as a proxy for the core genome. Each genome was searched using the sialidase protein alignment with HMMER (version 3.1b2). The scale bar indicates nucleotides substitutions per site.

A

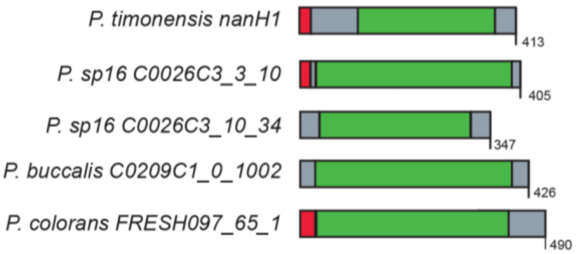

B

MUSCLE amino acid alignment					
*P. timonensis*_nanH1	*P. timonensis* nanH1	*P. sp16* C0026C3_3_10	*P. sp16* C0026C3_10_34	*P. buccalis* C0209C1_0_1002	*P. colorans* FRESH097_65_1
P. sp16 C0026C3_3_10	56.2				
P. sp16 C0026C3_10_34	22.8	20.1			
P. buccalis C0209C1_0_1002	61.6	54.9	20.2		
P. colorans FRESH097_65_1	9.5	10.6	10.6	9.5	

Figure S12. Predicted sialidase domains among new *Prevotella* sialidases from genome survey in Figure 4.
A) *Prevotella* encode several predicted sialidases with sialidase domains (green) and signal peptides (red). *P. timonensis* NanH1 was characterized in this study. B) MUSCLE alignment of proteins, the numbers represent % amino acid identity.

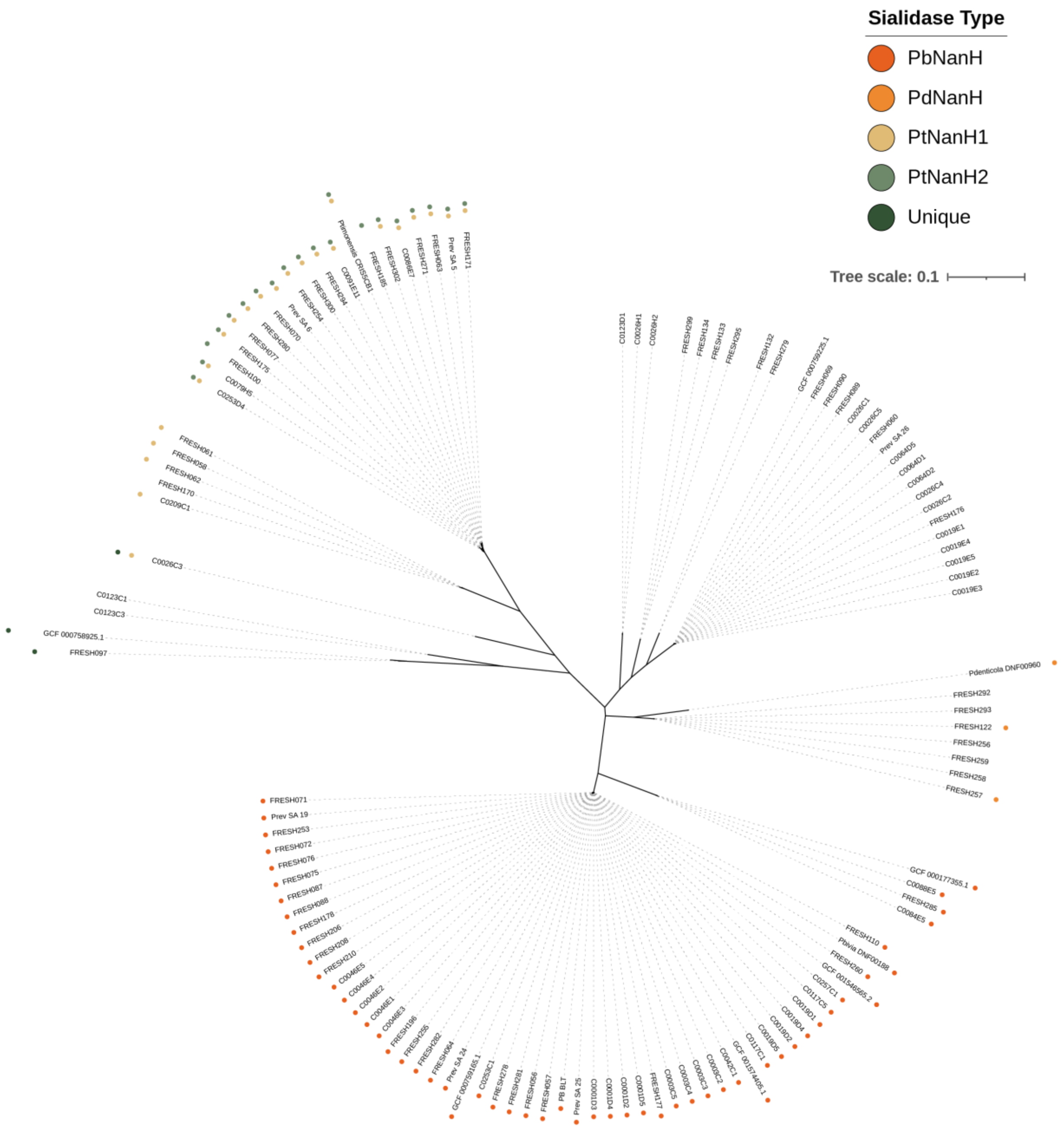

Figure S13. Unrooted phylogenetic trees of *Prevotella* vaginal isolates from the Vaginal Microbiome Research Consortium (N. American) and FRESH (S. African) studies.
This is the unrooted version of Figure 4A. The sialidase genes present in individual *Prevotella* isolate genomes are shown as filled circles. The phylogeny is based on 49 concatenated ribosomal proteins and serves as a proxy for the core genome. The phylogeny is based on 49 concatenated ribosomal proteins and serves as a proxy for the core genome. Each genome was searched using the sialidase protein alignment with HMMER (version 3.1b2).

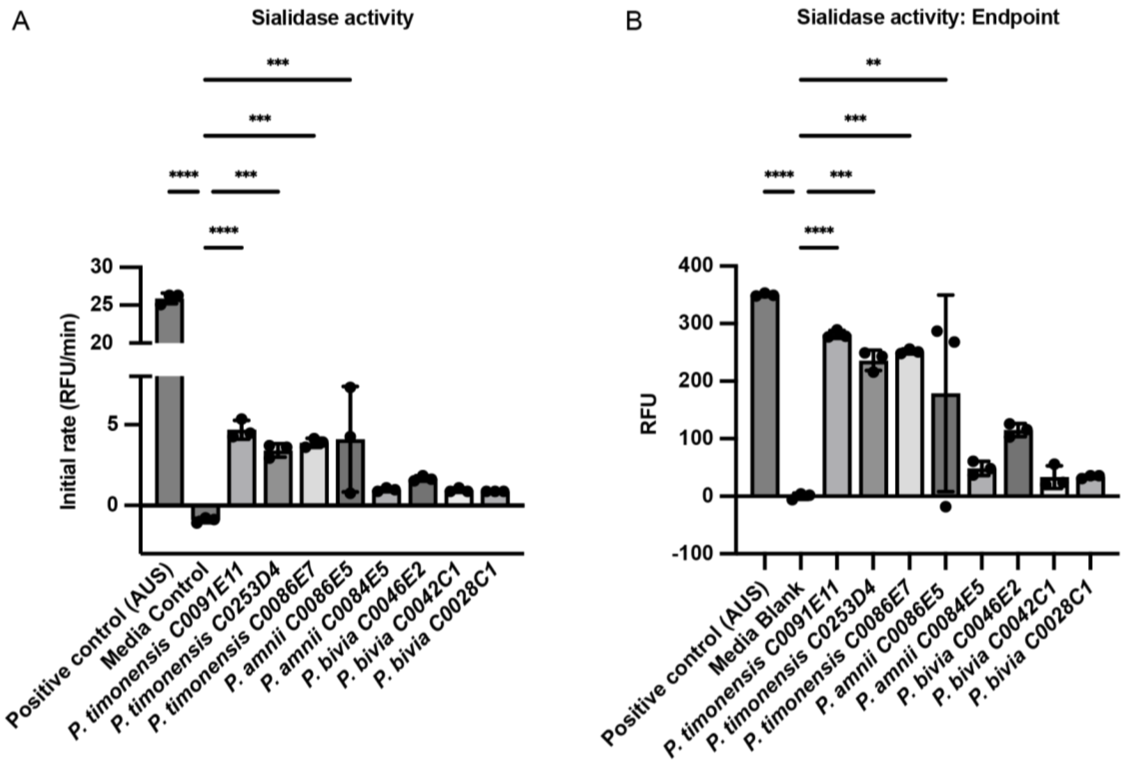

Figure S14. Multiple strains of vaginal *Prevotella* species possess sialidase activity in culture. Individual isolates of *Prevotella* species were cultured anaerobically for 48 hours in PYGT media containing 1% glucose and 10% horse serum. The cultures were then normalized to OD 1. Sialidase activity in culture was assayed in pH 5.5 sodium acetate buffer over 2 hours at 37 °C using 4-MU-Neu5Ac, at a final concentration of 350 µM. The positive control represents complete hydrolysis of the substrate by *Arthobacter ureafaciens* sialidase (AUS). The negative control is PYGT media. Data represent the average ± standard deviation of 3 biological replicates. Significance was assessed using one-way ANOVA followed by multiple comparisons test. Significance values represent comparison to the media control.

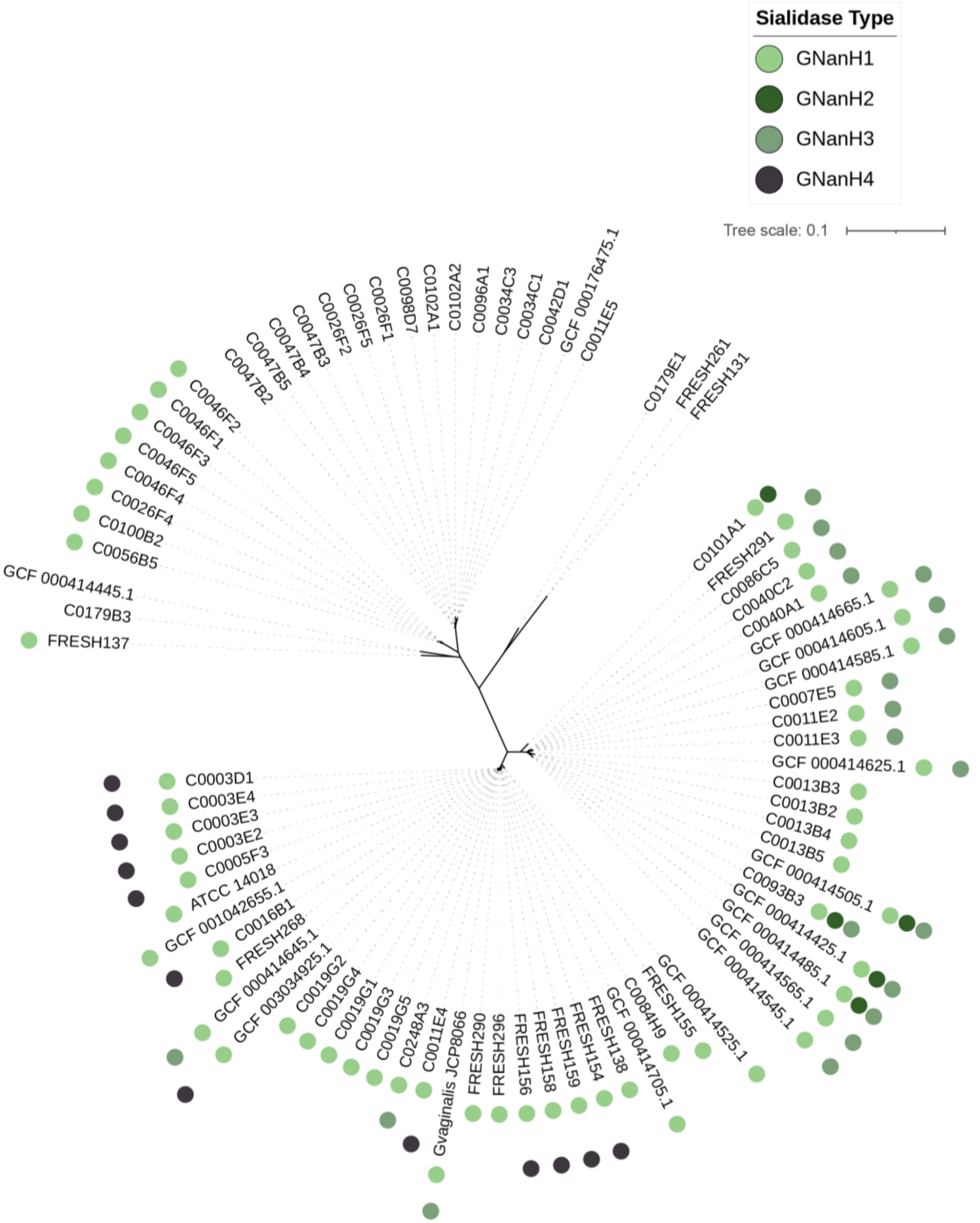

Figure S15. Unrooted phylogenetic trees of *Gardnerella* vaginal isolates from the Vaginal Microbiome Research Consortium (N. American) and FRESH (S. African) studies.
This is the unrooted version of Figure 4C. The sialidase genes present in individual *Gardnerella* isolate genomes are shown as filled circles. The phylogeny is based on 49 concatenated ribosomal proteins and serves as a proxy for the core genome. The phylogeny is based on 49 concatenated ribosomal proteins and serves as a proxy for the core genome. Each genome was searched using the sialidase protein alignment with HMMER (version 3.1b2).

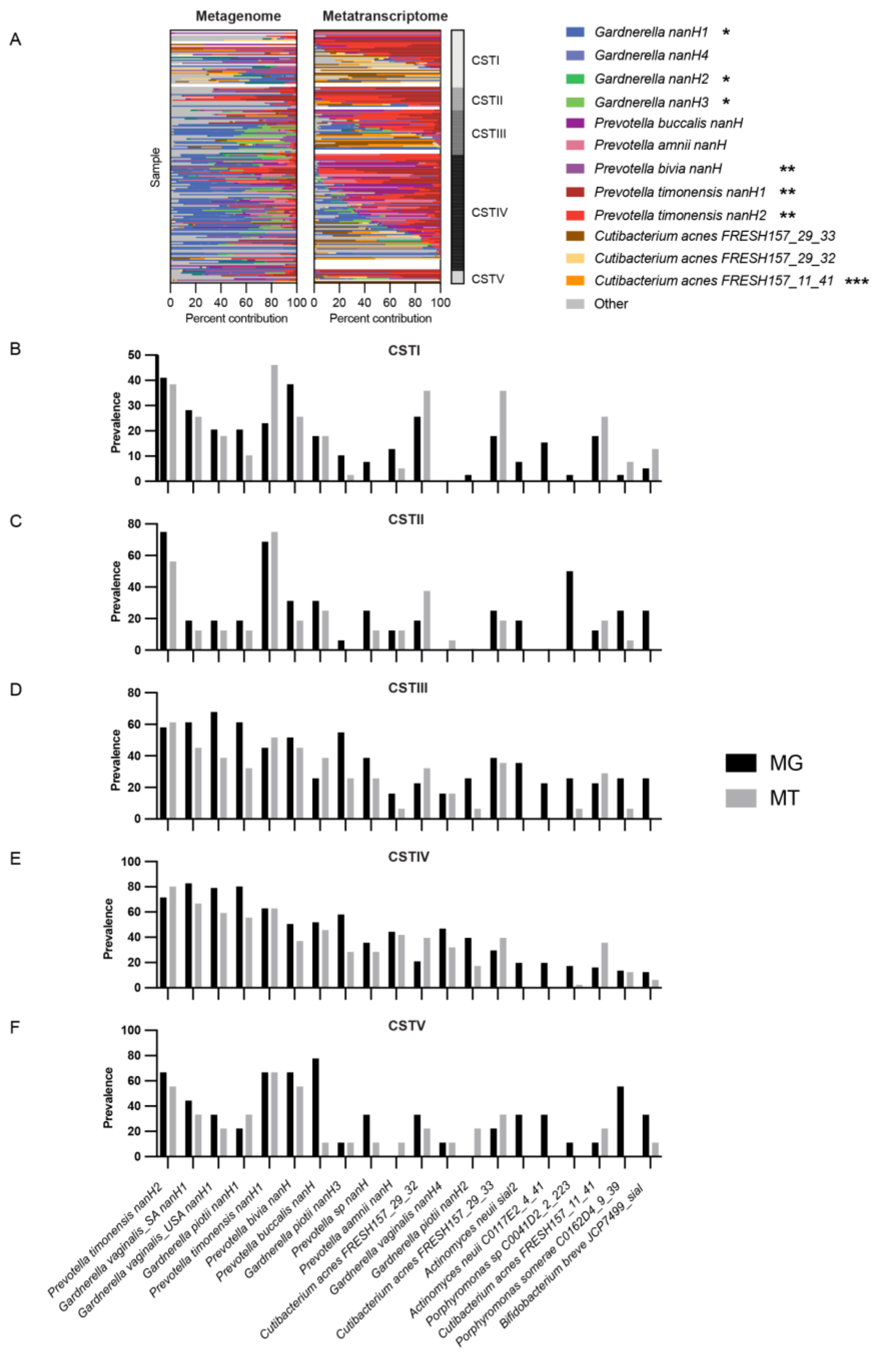

Figure S16. *Prevotella* sialidases are prevalent across all vaginal community state types. (A) Contribution of specific vaginal sialidase in metagenomes (left) and metatranscriptomes (right). Paired metagenomes (MG) and metatranscriptomes (MT) n=176 were used to investigate the relative contribution of several vaginal sialidases. * indicates enzymes were characterized previously [8], ** characterized in this study, *** indicates a *Cutibacterium acnes* sialidase with 99.6% a.a. ID to formerly characterized *Propionibacterium acnes* sialidase (PaNa, GenBank: ATT83304.1)[9]. This panel displays the same data in Figure 5D, in more detail to display the resolution of specific genes present in samples. Prevalence of select sialidase genes with >15 % prevalence in MG samples found in (B) CSTI (C) CSTII (D) CSTIII (E) CSTIV (F) CSTV. This panel displays the same data in Figure 5A, binned by community state type.

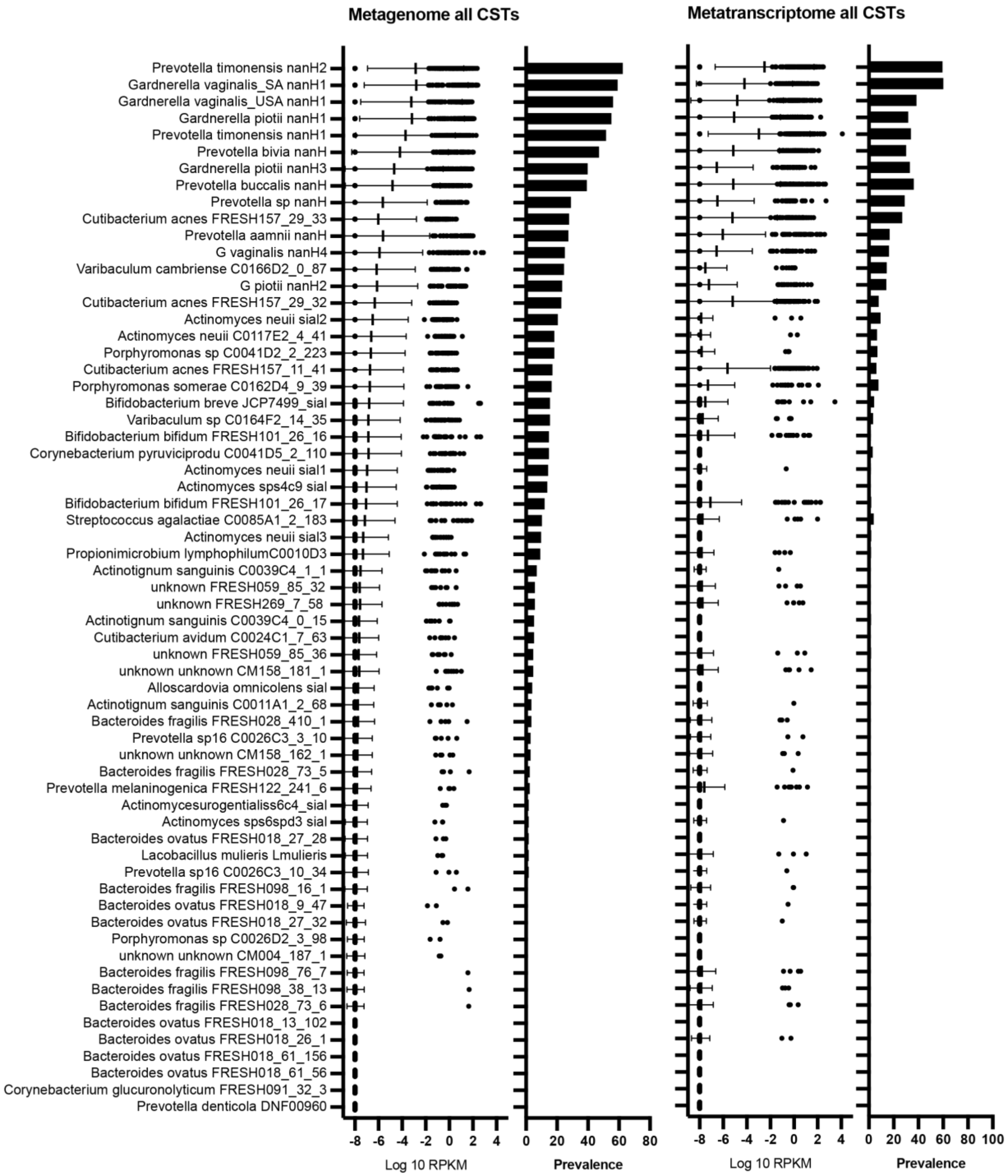

Figure S17. Sialidase gene abundance and prevalence across all CSTs.
The abundance and prevalence of specific sialidase genes in all paired MG and MT samples (n=176). This figure is the expanded version of Figure 5C-D. Abundance was determined by Diamond blastX and displayed as reads per kilobase million (RPKM). Figure displays all sialidase representative genes identified via the HMMER search from Figure 4. Abundance values were calculated by adding RPKM to a pseudo count (1E8), the bar represents the mean ± SD.

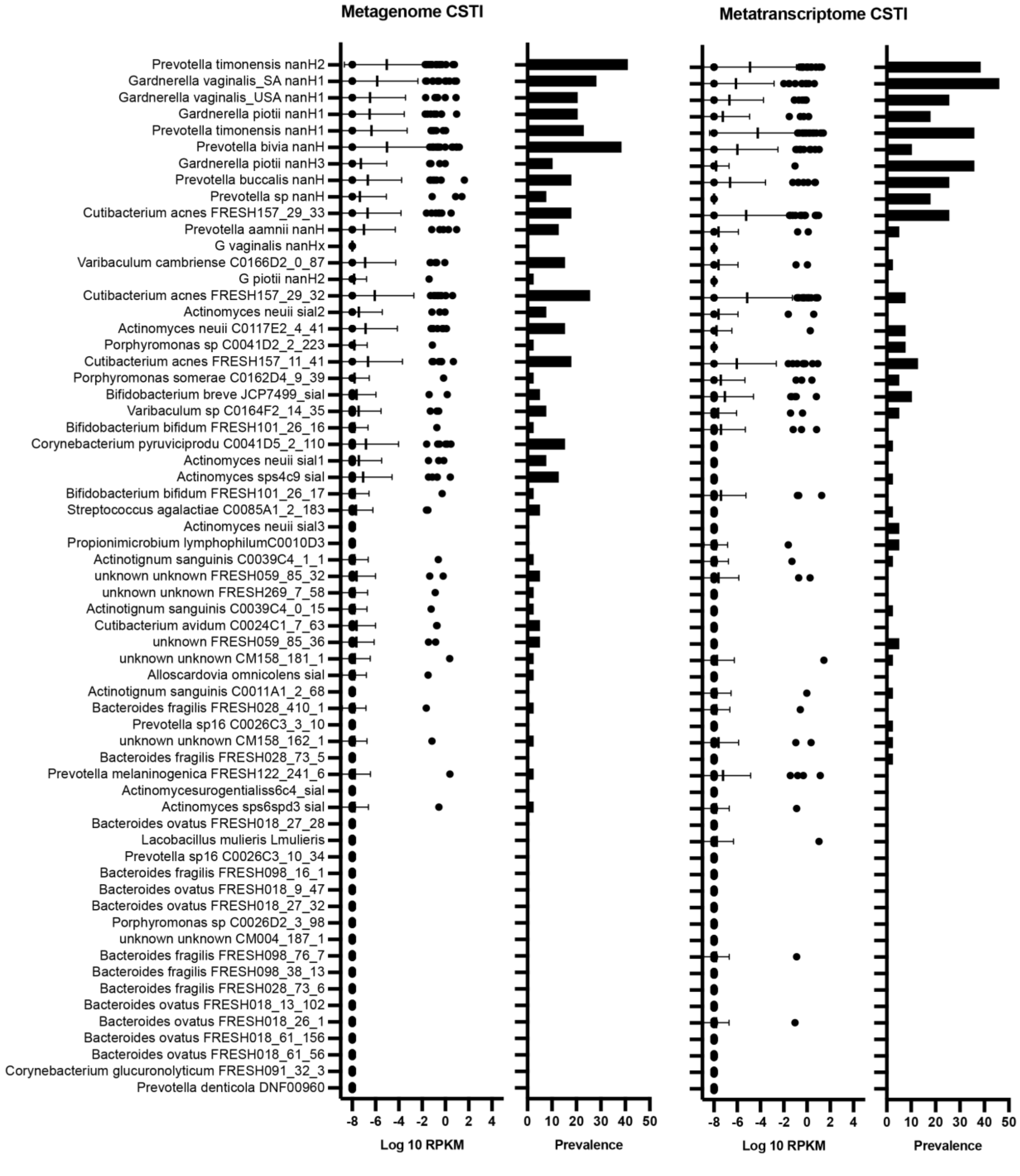

Figure S18. Sialidase gene abundance and prevalence across CSTI.

The abundance and prevalence of specific sialidase genes in paired CSTI MG and MT samples (n=39). Abundance was determined by Diamond blastX and displayed as reads per kilobase million (RPKM). Figure displays all sialidase genes identified via the HMMER search from Figure 4. Abundance values were calculated by adding RPKM to a pseudo count (1E8), the bar represents the mean ± SD.

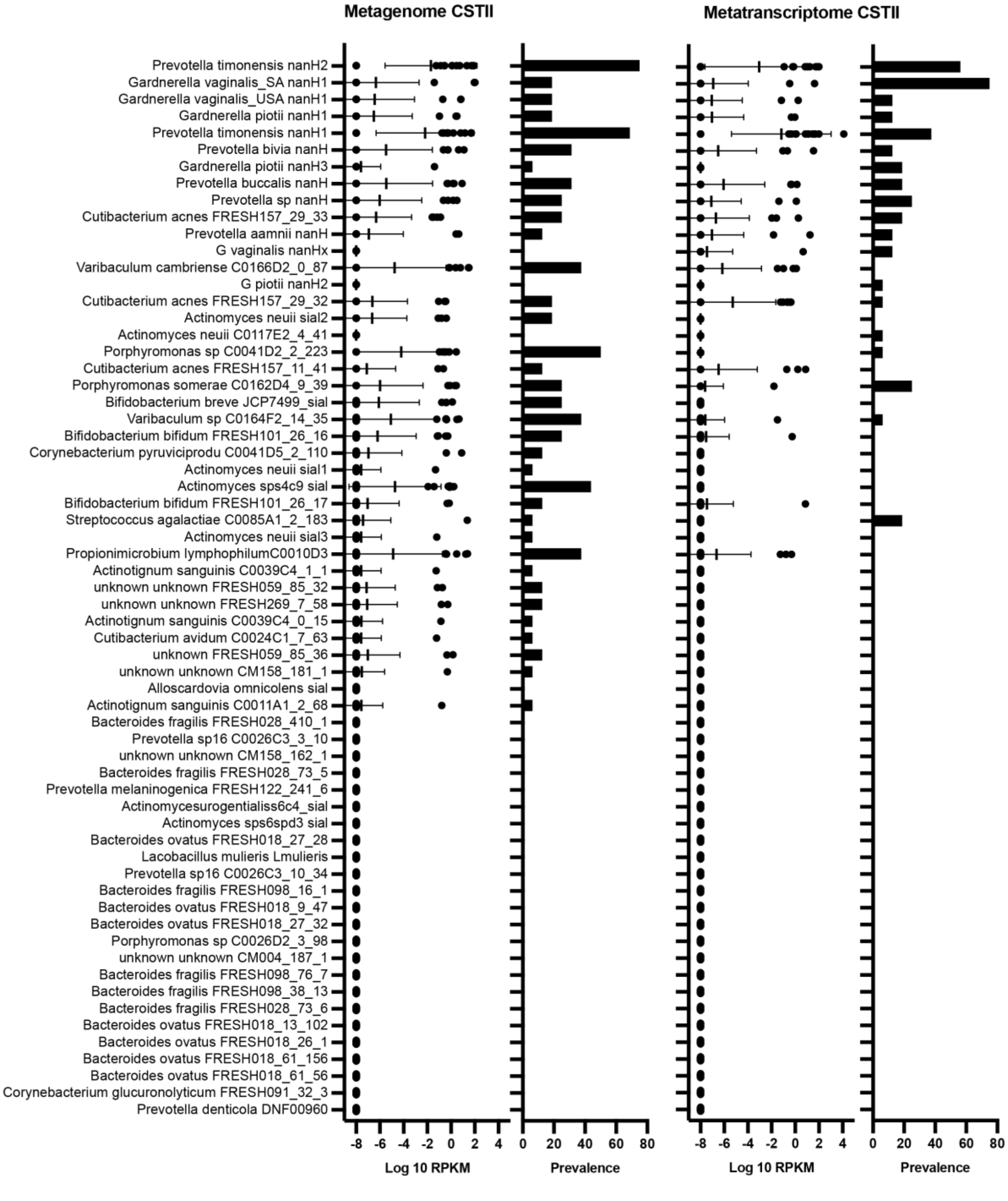

Figure S19. Sialidase gene abundance and prevalence across CSTII.
The abundance and prevalence of specific sialidase genes in paired CSTII MG and MT samples (n=16). Abundance was determined by Diamond blastX and displayed as reads per kilobase million (RPKM). Figure displays all sialidase genes identified via the HMMER search from Figure 4. Abundance values were calculated by adding RPKM to a pseudo count (1E8), the bar represents the mean ± SD.

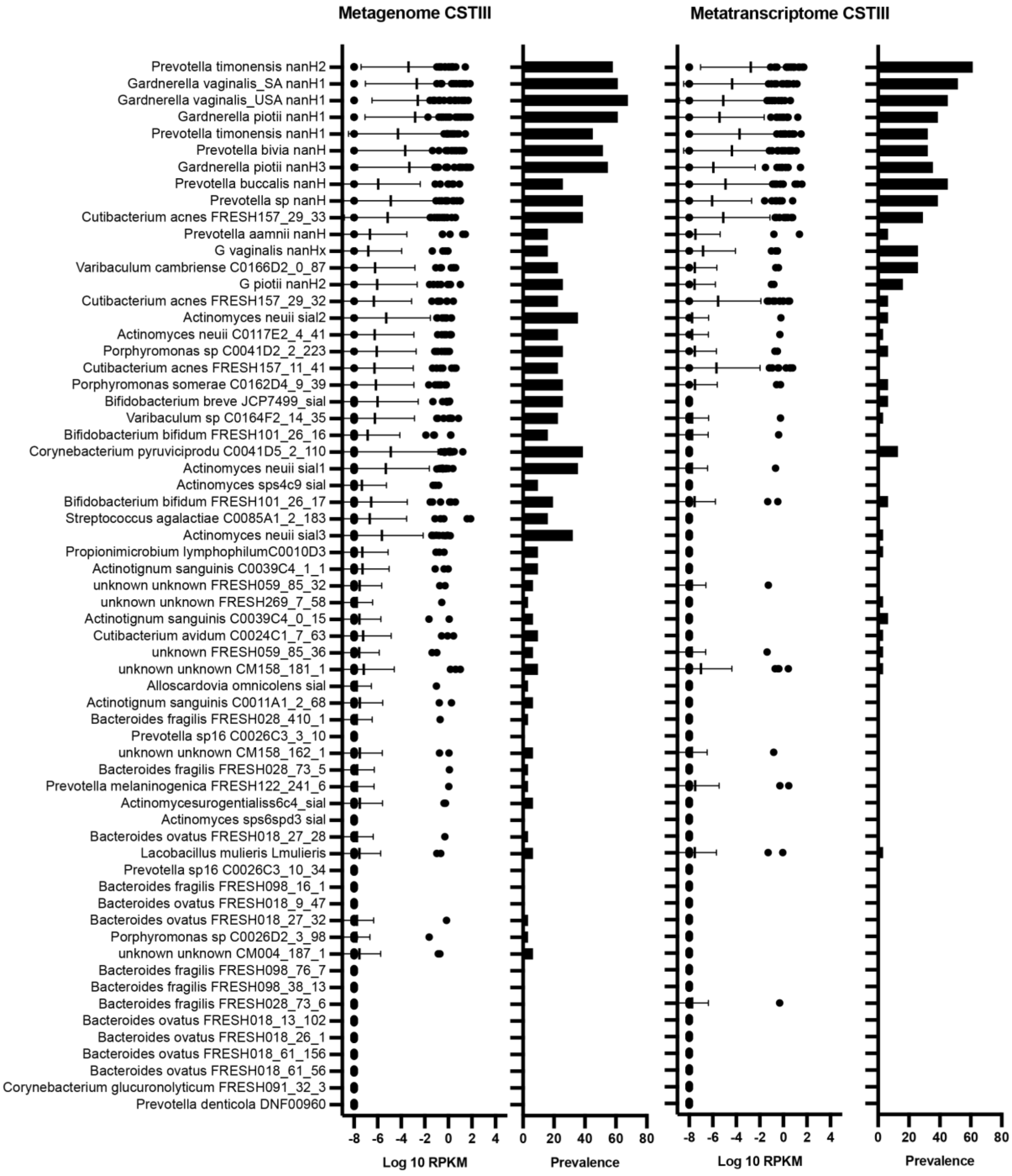

Figure S20. Sialidase gene abundance and prevalence across CSTIII.
The abundance and prevalence of specific sialidase genes in paired CSTIII MG and MT samples (n=31). Abundance was determined by Diamond blastX and displayed as reads per kilobase million (RPKM). Figure displays all sialidase genes identified via the HMMER search from Figure 4. Abundance values were calculated by adding RPKM to a pseudo count (1E8), the bar represents the mean ± SD.

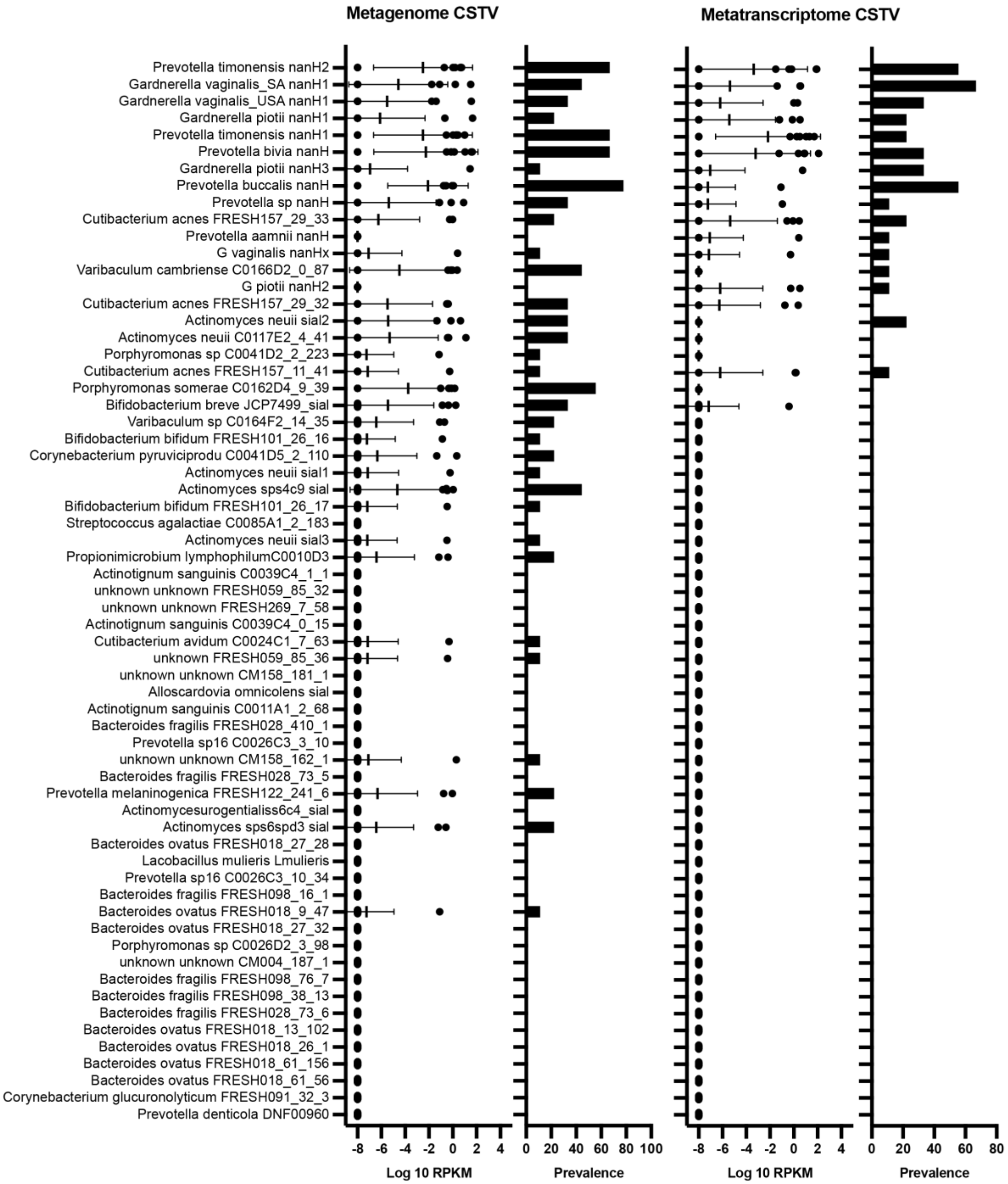

Figure S21. Sialidase gene abundance and prevalence across CSTIV.
The abundance and prevalence of specific sialidase genes in paired CSTIV MG and MT samples (n=81). Abundance was determined by Diamond blastX and displayed as reads per kilobase million (RPKM). Figure displays all sialidase genes identified via the HMMER search from Figure 4. Abundance values were calculated by adding RPKM to a pseudo count (1E8), the bar represents the mean ± SD.

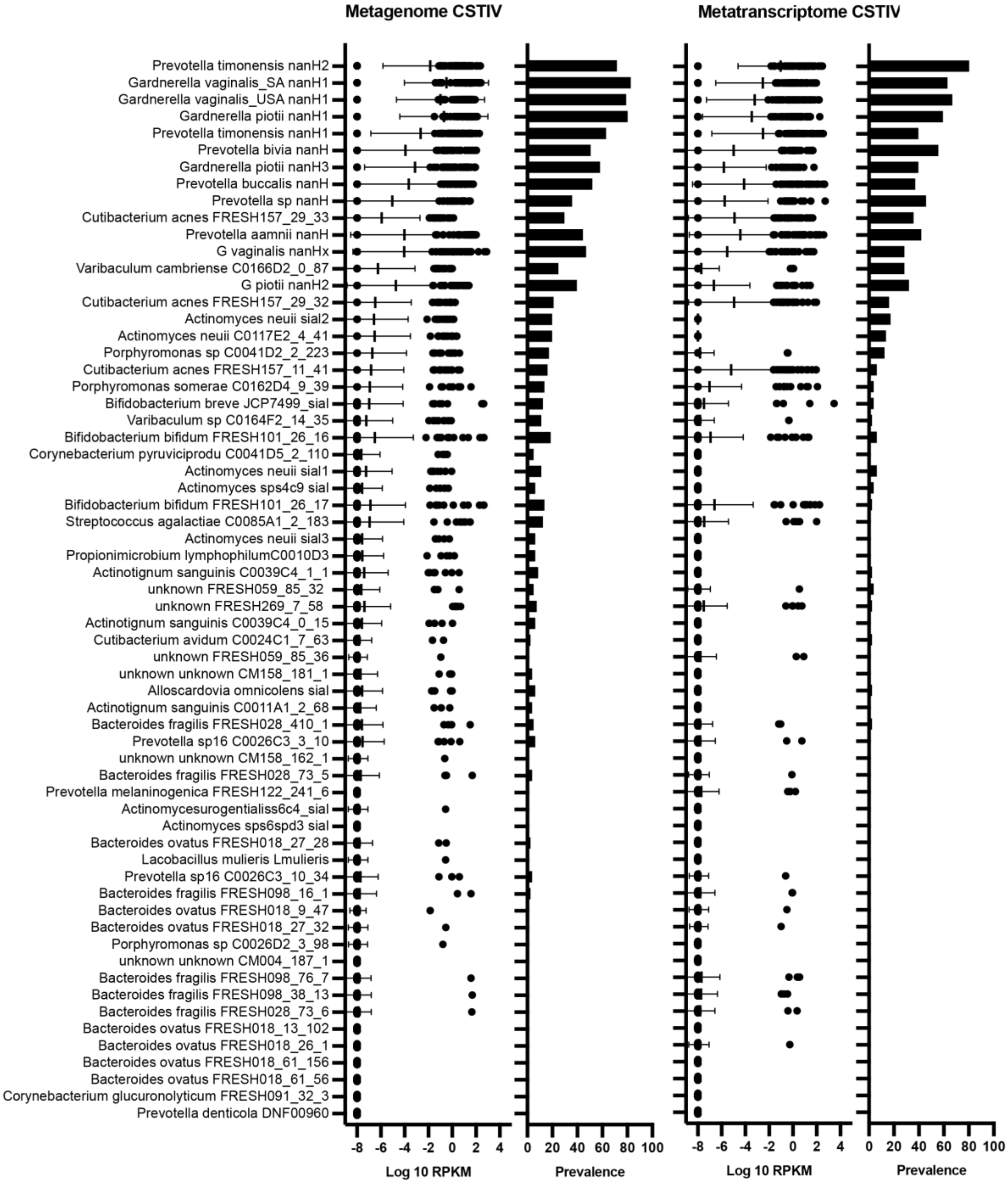

Figure S22. Sialidase gene abundance and prevalence across CSTV.
The abundance and prevalence of specific sialidase genes in paired CSTV MG and MT samples (n=9). Abundance was determined by Diamond blastX and displayed as reads per kilobase million (RPKM). Figure displays all sialidase genes identified via the HMMER search from Figure 4. Abundance values were calculated by adding RPKM to a pseudo count (1E8), the bar represents the mean ± SD.

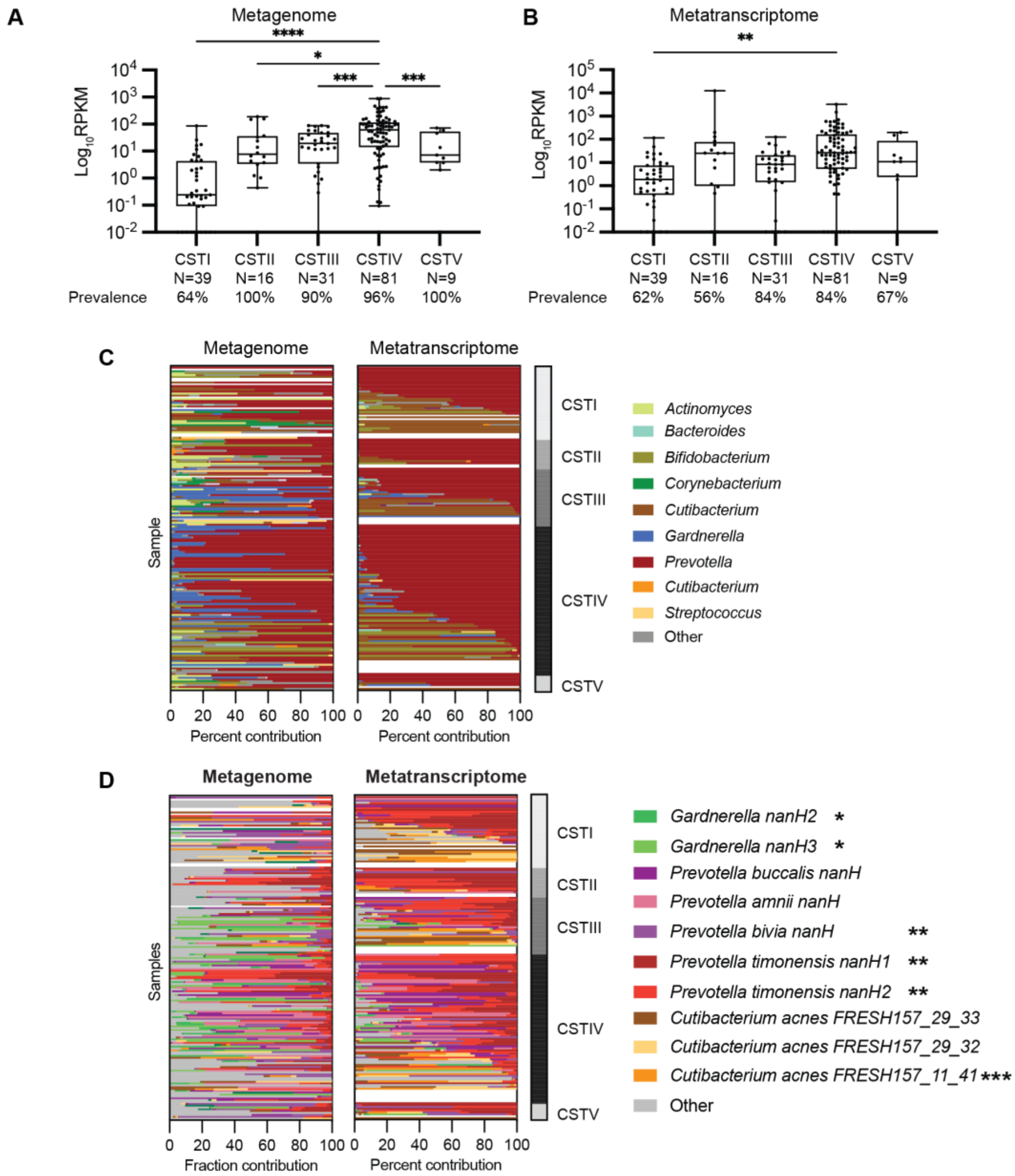

Figure S23. Analysis of vaginal metagenomes and metatranscriptomes for sialidase encoding genes excluding *Gardnerella nanh1* and *nanh4*.

(A) Total sialidase abundance and prevalence in paired metagenome and (B) metatranscriptomes samples (n=176). Abundance was determined by Diamond blastX and displayed as reads per kilobase million (RPKM). Significance was assessed by a one-way ANOVA followed by a Brown-Forsythe and Welch test, **p<0.0001, **p<0.01. All reads for *Gardnerella* nanh1 and nanh4 were removed prior to analysis. (C) Contribution of specific vaginal sialidase in metagenomes (left) and metatranscriptomes (right). Paired metagenomes (MG) and metatranscriptomes (MT) n=176 were used to investigate the relative contribution of several vaginal sialidases. All reads for *Gardnerella nanh1* and *nanh4* were removed prior to analysis. (D) This panel displays the same data in panel C, in more detail to display the resolution of specific genes present in samples. * indicates enzymes were characterized previously [8], ** characterized in this study, *** indicates a *Cutibacterium acnes* sialidase with 99.6% a.a. ID to formerly characterized *Propionibacterium acnes* sialidase (PaNa, GenBank: ATT83304.1)[9]. All reads for *Gardnerella nanh1* and *nanh4* were removed prior to analysis.

Supplemental References

1. Yu, A. C. Y. *et al.* Crystal structure of the Propionibacterium acnes surface sialidase, a drug target for P. acnes-associated diseases. *Glycobiology* **32**, 162–170 (2022).
2. Zaramela, L. S. *et al.* Gut bacteria responding to dietary change encode sialidases that exhibit preference for red meat-associated carbohydrates. *Nat Microbiol* **4**, 2082–2089 (2019).
3. Hayre, J. K. *et al.* Optimization of a direct spectrophotometric method to investigate the kinetics and inhibition of sialidases. *BMC Biochem* **13**, (2012).
4. Andersch-Björkman, Y., Thomsson, K. A., Holmén Larsson, J. M., Ekerhovd, E. & Hansson, G. C. Large Scale Identification of Proteins, Mucins, and Their *O* -Glycosylation in the Endocervical Mucus during the Menstrual Cycle. *Molecular & Cellular Proteomics* **6**, 708–716 (2007).
5. Werlang, C., Cárcarmo-Oyarce, G. & Ribbeck, K. Engineering mucus to study and influence the microbiome. *Nat Rev Mater* **4**, 134–145 (2019).
6. Werlang, C. A. *et al.* Mucin O-glycans suppress quorum-sensing pathways and genetic transformation in Streptococcus mutans. *Nat Microbiol* (2021) doi:10.1038/s41564-021-00876-1.
7. Kim, J. *et al.* Structural and quantitative characterization of mucin-type O-glycans and the identification of Oglycosylation sites in bovine submaxillary mucin. *Biomolecules* **10**, (2020).
8. Robinson, L. S., Schwebke, J., Lewis, W. G. & Lewis, A. L. Identification and characterization of NanH2 and NanH3, enzymes responsible for sialidase activity in the vaginal bacterium Gardnerella vaginalis. *Journal of Biological Chemistry* **294**, 5230–5245 (2019).
9. Yu, A. C. Y. *et al.* Crystal structure of the Propionibacterium acnes surface sialidase, a drug target for P. acnes-associated diseases. *Glycobiology* **32**, 162–170 (2022).
